# Small extracellular vesicles secreted from human amniotic fluid mesenchymal stromal cells possess cardioprotective and promigratory potential

**DOI:** 10.1007/s00395-020-0785-3

**Published:** 2020-03-07

**Authors:** Kaloyan Takov, Zhenhe He, Harvey E. Johnston, John F. Timms, Pascale V. Guillot, Derek M. Yellon, Sean M. Davidson

**Affiliations:** 1 The Hatter Cardiovascular Institute, University College London, 67 Chenies Mews, London, WC1E 6HX UK; 2 EGA Institute for Women’s Health, University College London, London, UK

**Keywords:** Exosomes, Foetal stem cells, Size-exclusion chromatography, Ischaemia–reperfusion injury, Angiogenesis, PI3K, Proteomics

## Abstract

Mesenchymal stromal cells (MSCs) exhibit antiapoptotic and proangiogenic functions in models of myocardial infarction which may be mediated by secreted small extracellular vesicles (sEVs). However, MSCs have frequently been harvested from aged or diseased patients, while the isolated sEVs often contain high levels of impurities. Here, we studied the cardioprotective and proangiogenic activities of size-exclusion chromatography-purified sEVs secreted from human foetal amniotic fluid stem cells (SS-hAFSCs), possessing superior functional potential to that of adult MSCs. We demonstrated for the first time that highly pure (up to 1.7 × 10^10^ particles/µg protein) and thoroughly characterised SS-hAFSC sEVs protect rat hearts from ischaemia–reperfusion injury in vivo when administered intravenously prior to reperfusion (38 ± 9% infarct size reduction, *p* < 0.05). SS-hAFSC sEVs did not protect isolated primary cardiomyocytes in models of simulated ischaemia–reperfusion injury in vitro, indicative of indirect cardioprotective effects. SS-hAFSC sEVs were not proangiogenic in vitro, although they markedly stimulated endothelial cell migration. Additionally, sEVs were entirely responsible for the promigratory effects of the medium conditioned by SS-hAFSC. Mechanistically, sEV-induced chemotaxis involved phosphatidylinositol 3-kinase (PI3K) signalling, as its pharmacological inhibition in treated endothelial cells reduced migration by 54 ± 7% (*p* < 0.001). Together, these data indicate that SS-hAFSC sEVs have multifactorial beneficial effects in a myocardial infarction setting.

**Electronic supplementary material:**

The online version of this article (10.1007/s00395-020-0785-3) contains supplementary material, which is available to authorized users.

## Introduction

The term “extracellular vesicles” (EVs) encompasses various populations of lipid-bilayer, nanosized vesicles released into the extracellular space by all cell types [42]. With their diverse protein and RNA cargo, small EVs (sEVs, 30–150 nm in diameter; commonly referred to as “exosomes” [33, 58]) represent an exciting, potentially multitarget therapeutic for cardioprotection and heart repair [16, 17, 20, 52]. sEVs can act as a means of communication between cells and may be responsible for a plethora of physiological or pathophysiological processes in the cardiovascular system [52].

Multipotent mesenchymal stromal/stem cells (MSCs) have been well documented to aid cardiac repair and long-term functional improvement of the heart following myocardial infarction [2]. These effects appear to be conferred by the MSC secretome [59], and are at least partially mediated by secreted sEVs [34, 52]. Multipotent MSCs can be isolated throughout development from a range of foetal as well as adult tissues [27, 28]. Compared to their adult counterparts, foetal MSCs possess greater expansion capacity [27] as well as better functional potential than adult MSCs [4, 48]. Amniotic fluid represents a rich source of foetal MSC, i.e., amniotic fluid stem cells (AFSCs), which can be harvested without significant ethical concerns during routine amniocenteses [27, 28, 62]. Both human AFSCs (hAFSCs) and hAFSC-conditioned medium alone were shown to decrease infarct size in a rat model of acute ischaemia–reperfusion injury [9], while hAFSCs are also more effective than adult bone marrow MSCs in promoting capillary formation in vivo [48]. The study of hAFSC EVs has only commenced recently, and their functional effects are not well understood [4, 6]. Limited experimentation indicates that hAFSC sEVs may not be potently proangiogenic in their naïve state [5, 6]. On the other hand, Balbi et al*.* have recently demonstrated that hAFSC sEVs can reduce infarct size and improve cardiac function when injected intramyocardially in a model of permanent myocardial infarction in mice [5]. It remains unknown whether hAFSC sEVs deliver cardioprotective or proangiogenic benefits in a setting of myocardial ischaemia–reperfusion.

Amongst the different hAFSC subpopulations, spindle-shaped fibroblast-like hAFSCs (SS-hAFSCs) represent only a small fraction of the hAFSCs, but exhibit considerably greater stemness and protective potential than other hAFSC subpopulations, as demonstrated in a model of brain ischaemia in mice [14]. Thus, SS-hAFSCs are an attractive and unexplored novel source of secreted factors, which may have potential clinical application in the treatment of myocardial infarction and its consequences.

A major debate in the sEV field concerns the purity of the obtained vesicle samples due to contamination of the samples with non-EV proteins, RNAs, and lipoproteins [42, 58]. Furthermore, common and widely used isolation approaches, such as ultracentrifugation, may compromise sEV integrity leading to vesicle breakdown, aggregation, or fusion which can alter the functions of the sEVs [35]. On the other hand, size-exclusion chromatography (SEC) is a technique deemed to be superior to most other methods as it does not require high-speed centrifugation, lengthy procedures, or expensive equipment, and it preserves vesicular structure well [8, 37].

In this report, we isolated sEVs from SS-hAFSC-conditioned medium using a highly standardised protocol of ultrafiltration combined with SEC. We systematically characterised the purified vesicles and investigated their role in cardioprotection and angiogenesis. Our findings demonstrated that SS-hAFSC sEVs provide acute protection to the rat myocardium against ischaemia–reperfusion injury in vivo when administered via a clinically suitable intravenous route at the time of reperfusion. Intriguingly, sEVs did not protect isolated rat cardiomyocytes in vitro, indicative of indirect pro-survival effects. SS-hAFSC were not proangiogenic in vitro but promoted marked migration of endothelial cells which required phosphatidylinositol-3-kinases (PI3K) signalling. We have further shown that sEVs are an essential component of the SS-hAFSC-conditioned medium which loses its promigratory activity upon sEV depletion. These findings shed new light on the benefits provided by foetal MSC sEVs in cardioprotection and angiogenesis.

## Materials and methods

Extended procedural details are provided in the Supplementary Methods.

### Cell culture

Amniotic fluid (AF) was collected from healthy donors after written informed consent was obtained from all participants or their legal guardians, in compliance with the Declaration of Helsinki. The ethical approval was given by the Research Ethics Committees of Hammersmith & Queen Charlotte’s Hospitals (2001/6234) for frozen samples and from NRES Committee London, Bloomsbury (14/LO/0863) for fresh samples, in compliance with UK national guidelines [Review of the Guidance on the Research Use of Fetuses and Fetal Material (1989) also known as Polkinghorne Guideline. London: Her Majesty’s Stationery Office, 1989: Cm762)] for the collection of human foetal tissue for research.

SS-hAFSCs were isolated as described previously [14]. Briefly, amniotic fluid was spun at low speed to pellet the cells which were resuspended in complete medium, seeded, and incubated until the appearance of adherent fibroblast-like cells. Colonies with similar morphology (i.e., spindle-shaped) were propagated and characterised for MSC markers. SS-AFSCs were maintained in monolayers in DMEM (ThermoFisher) supplemented with 25-mM glucose, 4-mM GlutaMAX, 50 units/ml penicillin (Sigma), 50-µg/ml streptomycin (Sigma), and 9% FBS (F9665, Sigma). SS-AFSCs from passages 11–20 were used for experiments.

Human umbilical vein endothelial cells (HUVECs) were obtained from Lonza as a pooled donor sample (C2519A) and maintained in monolayers in Endothelial Cell Basal Medium 2 (C-22211, PromoCell) supplemented with Endothelial Cell Growth Medium 2 SupplementPack (C-39211, PromoCell). HUVECs from passages 5–11 were used for experiments.

SS-hAFSCs and HUVECs were grown in a conventional tissue culture incubator at 37 °C/5% CO_2_ and detached with TrypLE Express Enzyme (ThermoFisher) for passaging. SS-hAFSCs images were taken using Nikon Eclipse TE200 inverted microscope (Nikon).

### Flow cytometry

SS-hAFSC were characterised by flow cytometry using the antibodies listed in Supplementary Table 1. See supplementary methods for further details.

SS-hAFSCs’ death and apoptosis were estimated by Apoptosis Detection Kit I (556547, BD Biosciences) following the manufacturer’s instructions.

### Isolation of SS-hAFSC small extracellular vesicles (sEVs)

SS-hAFSC-conditioned medium was collected from ~ 5–10 × 10^6^ SS-AFSCs (2 × T225 flasks, 60 ml culture medium in total) cultured in serum-free conditions for the indicated time periods. SS-AFSC sEVs for tube formation assay and in vivo experiments were isolated from ~ 40–55 × 10^6^ SS-AFSCs (10 × T225 flasks, 300 ml culture medium in total). Conditioned medium was spun at 300 g for 10 min, 4 °C to remove dead cells. Supernatant was then centrifuged at 10,000 g for 40 min, 4 °C (polycarbonate tubes, 355,630, Beckman Coulter; MLA-55 rotor, Optima MAX-XP, Beckman Coulter) to remove cell debris and large vesicles. After discarding the pellet, conditioned medium was concentrated to ~ 200–450 µl using Vivaspin-15R ultrafiltration units (30 kDa, Hydrosart membrane, Sartorius). The remaining concentrate was immediately processed for SEC on qEVoriginal columns (iZON Science) [8]. Fractions were collected and pooled as indicated and stored at − 80 °C.

### Characterisation of SS-hAFSC small extracellular vesicles (sEVs)

#### Nanoparticle tracking analysis

Nanoparticle tracking analysis (NTA) was performed following recommendations [26] adapted to the type of samples in our studies. NanoSight LM10-HS instrument (Malvern), 488-nm laser module, and NTA 3.1 software version were used for analysis. A syringe pump with constant flow injection was used and 3–5 videos of 30 s were captured with Camera Level of 15 and Detection Threshold of 4.

#### Protein assays

Bicinchoninic acid (BCA) protein assay kit for low concentrations (ab207002, Abcam) and Bradford assays (in-house made using Coomassie Brilliant Blue G-250, 44,329, BDH) were used to quantify the protein content of SS-hAFSC-conditioned medium and sEV isolates. BCA assays were performed according to the manufacturer’s instructions (300-µl final reaction volumes; 2-h incubation time). Higher protein content samples were analysed using standard BCA assay (Sigma) in 200-µl final reaction volumes for a 30-min incubation time. Absorbance for both assays was read at 562 nm on an FLUOstar plate reader (BMG Labtech) and protein concentrations were calculated using BSA standards and a four-parameter logistic curve.

Bradford assays were performed in 300-µl final reaction volumes, with 10-min incubation time and measurement of absorbance at 595 nm on an FLUOstar plate reader (BMG Labtech). Protein concentrations were calculated using BSA standards and a four-parameter logistic curve.

#### Dot blot protein analysis

~ 50-ng protein of SS-hAFSC lysate, SS-hAFSC-conditioned medium, or isolated SS-hAFSC sEVs was lysed using 0.1% (v/v) Triton X-100 (Sigma), vortexed, and pipetted on nitrocellulose membranes (10,600,003, GE Healthcare). After drying out, the membranes were blocked using a solution of 5% BSA/0.1% Tween-20 (Sigma) in PBS for 1 h at room temperature, and incubated with primary antibodies at 1 μg/ml in 5% BSA/0.1% Tween-20/PBS overnight at 4 °C (CD63: Clone H5C6, BD Biosciences; CD81: Clone JS-81, BD Biosciences; ACTN4: Clone C2C3, GeneTex). Secondary antibodies were added for 1 h at room temperature (1/10,000; goat anti-mouse IgG for CD63 and CD81, 926–32,210, LI-COR and goat anti-rabbit IgG for ACTN4, 926–32,211, LI-COR). Membranes were imaged using Odyssey system (LI-COR).

#### Dissociation-enhanced lanthanide fluorescence immunoassay (DELFIA)

Exosome-specific markers were quantified using a previously described dissociation-enhanced lanthanide fluorescence immunoassay (DELFIA) [55, 56, 64]. Samples were added to a high-binding 96-well microplate (DY990, R&D Systems). After overnight incubation at 4 °C, blocking with 1% BSA/PBS for 1 h at room temperature was performed. This was followed by primary antibody incubation at 1 μg/ml in PBS for 2 h at room temperature (CD9: Clone M-L13, BD Biosciences; CD63: Clone H5C6, BD Biosciences; CD81: Clone JS-81, BD Biosciences) and secondary antibody incubation at 0.25 µg/ml in PBS for 1 h at room temperature (biotin-conjugated goat anti-mouse IgG1, ab98691, Abcam). 1:1000 Eu-labelled streptavidin in DELFIA Assay Buffer (PerkinElmer) was then added and incubated for 1 h at room temperature. Finally, 100-µl DELFIA Enhancement Solution (PerkinElmer) was added to each well and time-resolved fluorimetry was performed using a PHERAstar plate reader (BMG Labtech) with excitation at 337 nm, detection at 620 nm, integration start at 60 µs, and integration time of 200 µs. Results are presented as arbitrary units (AU).

#### Transmission electron microscopy (TEM)

~ 2 µl of each sample were adsorbed on Formvar-carbon electron microscopy grids. After washing with H_2_O, the grids were transferred to a drop of 0.5% uranyl acetate solution [57], pH 7 for ~ 3 min. Excess fluid was blotted, and the grids were imaged using a Jeol JEM-1010 electron microscope (Jeol Ltd).

#### Protein arrays

Protein profiling of SS-hAFSC sEVs was performed using Proteome Profiler Human Angiogenesis Array Kit and Cytokine Array Kit (ARY007 and ARY005B, respectively; R&D Systems) following the manufacturer’s instructions with some modifications. Each membrane was incubated with ~ 15 µg SS-hAFSC sEV protein for Cytokine Arrays and ~ 10 µg SS-hAFSC sEV protein for Angiogenesis Arrays. Prior to incubation, sEVs were lysed with addition of 0.1% (v/v) Triton X-100 and vortexing for 30 s [43]. Streptavidin-DyLight 800 conjugate was used for the detection of biotin-conjugated antibodies at 250 ng/ml (21,851, ThermoFisher). Membranes were imaged, and densitometry was performed on Odyssey system. The spot coordinates can be found on https://resources.rndsystems.com/pdfs/datasheets/ary007.pdf and https://resources.rndsystems.com/pdfs/datasheets/ary005b.pdf. Duplicate spot pixel densities were normalised to reference control spots and presented as relative pixel densities.

#### Proteomics: liquid chromatography–tandem mass spectrometry (LC–MS/MS)

SS-hAFSC-conditioned medium or isolated SS-hAFSC sEVs containing ~ 30 µg of protein were lysed using 0.5% (w/v) sodium dodecyl sulphate and vortexing, and concentrated to ~ 50 µl on Vivaspin-500 ultrafiltration units (5-kDa cut-off membranes, Sartorius). Samples were then reduced using 200 mM *tris*(2-carboxyethyl)phosphine for 1 h at 60 ˚C, alkylated with 200-mM methyl methanethiosulfonate for 10 min at room temperature, and digested overnight with 1 µg of proteomics grade trypsin (Promega). Detergent was removed using a detergent removal column (ThermoFisher), and peptides were isolated and fractionated by solid-phase extraction using 100 µl C18 tips eluting into 5, 10, 15, 20, 25, 30, 35, 40, and 60% acetonitrile in 0.1% ammonium hydroxide. The peptide fractions were lyophilised and resuspended in 0.1% formic acid. ~ 500 ng of each fraction was loaded by Dionex Ulitmate 3000 (ThermoFisher) onto a Pepmap100 C18 trapping cartridge (5 mm × 5 µM × 0.3 µm) and eluted over a reverse phase gradient [6% (3 min), 35% (35 min), 90% (40 min) organic phase (95% ACN, 5% DMSO, 0.1% FA) in aqueous phase (5% DMSO, 0.1% FA)]. Peptides were resolved with a Pepmap C18 (25 cm × 3 µm × 100 Å) at 300 nl/min and analysed with an LQT-Orbitrap XL mass spectrometer (ThermoFisher). MS analysis of eluting peptides was conducted between 400 and 1700 m/z at 60,000 mass resolution. The top four precursor ions per MS scan were characterised by tandem MS with CID (ion trap MS, 2 Da isolation window, 35 keV). The DMSO ion at 401.922718 was used as a lockmass. Target-decoy searching of raw spectral data was performed with Proteome Discoverer software version 1.4.1.14 (ThermoFisher). Spectra were searched using SequestHT (version 1.1.1.11) against the human and bovine UniProt Swissprot database (downloaded July 2019; supplemented with cRAP contaminations). Settings were: a fragment ion mass tolerance of 0.5 Da, a precursor mass tolerance of 10 ppm, searching for tryptic peptides allowing one missed cleavage, fixed modification of Methythio (C), variable modification of oxidation (M), and deamidation (N,Q) with percolator used to estimate FDR with a threshold of *q* < 0.01.

Label-free quantitation (LFQ) enrichment ratios were calculated by LFQ of a 2-ppm precursor ion area and were normalised on total protein observation. The minimum LFQ peptide area value (defined as the smallest observed peptide quantitation) was 1.64E + 04. For minimum LFQ peptide area values, enrichment ratios are presented as “∞” or “− ∞” indicating protein detection only in sEVs or conditioned medium, respectively.

Peptide-spectrum match (PSM) enrichment ratios were calculated by determining the relative number of PSMs observed for each protein after normalisation on total protein observation.

Functional enrichment analysis was performed using g:Profiler (version e96_eg43_p13_563554d, database updated on 05/06/2019) with g:SCS multiple testing correction method and a significance threshold of 0.05 [46]. Proteins detected in the sEV samples or proteins enriched in or exclusive to the sEV samples were used, excluding those assigned a minimum LFQ value and those with < 2 PSMs. Fold enrichment analysis was performed which represents the occurrence of the proteins associated with the indicated terms relative to the expected occurrence based on the query size and the total human proteome size. Complete analysis can be found in the Supplementary Proteomics Table.

STRING database online tool version 11.0 [54] was used to produce a network of SS-hAFSC sEV-enriched and SS-hAFSC sEV-exclusive proteins (the highest confidence setting was chosen with interaction score of ≥ 0.900 and disconnected nodes were hidden). sEV enrichment was defined as > 1.5 times (log_2_ > 0.58) higher LFQ peptide area in the sEV sample compared to the conditioned medium sample.

Full data of the identified proteins are presented in the Supplementary Proteomics Table. “CM” notation in the table refers to SS-hAFSC-conditioned medium, while “sEVs” notation refers to SEC-isolated SS-hAFSC sEVs. “sEV-enr > 1.5x” refers to proteins enriched in sEVs > 1.5 times or exclusive to sEVs by LFQ peptide area.

#### EV-TRACK

The relevant data were submitted to the EV-TRACK knowledgebase (EV-TRACK ID: EV190058) [22].

### Ethical approval for animal use

All procedures were approved by the Animal Welfare and Ethical Review Body (AWERB) and were conducted within the terms of the United Kingdom Home Office Guide on the Operation of Animals (Scientific Procedures) Act 1986, the Directive 2010/63/EU of the European Parliament on the protection of animals used for scientific purposes and the NIH guidelines.

### In vivo non-recovery ischaemia–reperfusion injury model

Rats were anaesthetized with 100-mg/kg pentobarbital injected intraperitoneally, and then subjected to in vivo myocardial ischaemia–reperfusion injury by ligation of left anterior descending (LAD) artery for 30 min. Treatments [vehicle—PBS, bradykinin (positive control), SS-hAFSC sEVs] were administered intravenously via a jugular vein cannula 2 min prior to reperfusion. Following a 2-h reperfusion, experiments were terminated by excision of the heart, and myocardial infarct size was measured using tetrazolium staining [10, 36].

The in vivo experiments were randomised, and the operator was blinded to the administered treatment. The results are represented as the average of the analyses of two different experimenters who were blinded to treatment.

See supplementary methods for further details.

### Models of cardiomyocyte death in vitro

Primary cardiomyocytes were isolated from the left ventricles of buffer-perfused adult rat hearts digested with collagenase and protease. Cells were seeded on laminin pre-coated 24-well plates.

A model of acute reactive oxygen species (ROS)-induced death of cardiomyocytes was established by 2-h treatment of rat cardiomyocytes with hydrogen peroxide (H_2_O_2_).

A model of hypoxia/reoxygenation of rat cardiomyocytes in vitro was set up to simulate ischaemia–reperfusion injury in vitro (simulated ischaemia–reperfusion injury). Cardiomyocytes were subjected to hypoxia for 5 h followed by a reoxygenation for 1 h in hypoxic and normoxic buffers (Supplementary Table 2), respectively, to mimic normal and ischaemic milieu found in the heart during myocardial infarction.

The protective effects of SS-hAFSC sEVs in these models were evaluated using lactate dehydrogenase (LDH) release assay as a surrogate measurement for cell death.

See supplementary methods for further details.

### Endothelial cell assays

A modified Boyden’s chamber [11] assay was performed to assess for promigratory functions of SS-hAFSC sEV isolates on HUVECs.

HUVEC proliferation over a 48-h period was studied by 3-(4,5-dimethylthiazol-2-yl)-2,5-diphenyltetrazolium bromide (MTT) assay [41].

HUVEC tube formation assay on a thin-layer extracellular matrix-mimicking gel [24] was conducted to investigate the proangiogenic effects of SS-hAFSC sEVs in vitro.

See supplementary methods for further details.

### Studies of signalling pathways in endothelial cells

Activation of the PI3K signalling pathway (AKT and PRAS40) in HUVECs by SS-hAFSC sEVs was studied by western blotting (see Supplementary Table 3 for antibody details).

Phosphorylation of intracellular signalling kinase pathways was investigated using Proteome Profiler Human Phospho-Kinase Array Kit (ARY003B, R&D Systems).

See supplementary methods for further details.

### Statistical analysis

Data are plotted as means ± SEM. GraphPad Prism was used for statistical analyses and graph production (GraphPad Software). Statistical comparisons were performed using Student’s *t *tests, one-way ANOVA with Tukey’s or Dunnett’s post hoc tests or two-way ANOVA as indicated. *p* values of less than 0.05 were considered significant.

## Results

### Characterisation of SS-hAFSCs

Human SS-hAFSCs have previously been thoroughly characterised for their tri-lineage differentiation potential [40]. SS-hAFSCs expressed cell surface markers CD73 (99.3%), CD90 (65.3%), CD105 (99.5%), CD29 (99.1%), and CD44 (100.0%), and were negative for CD14, CD34, and CD45, satisfying the criteria for MSC identification [23] (Supplementary Fig. 1).

**Fig. 1:**
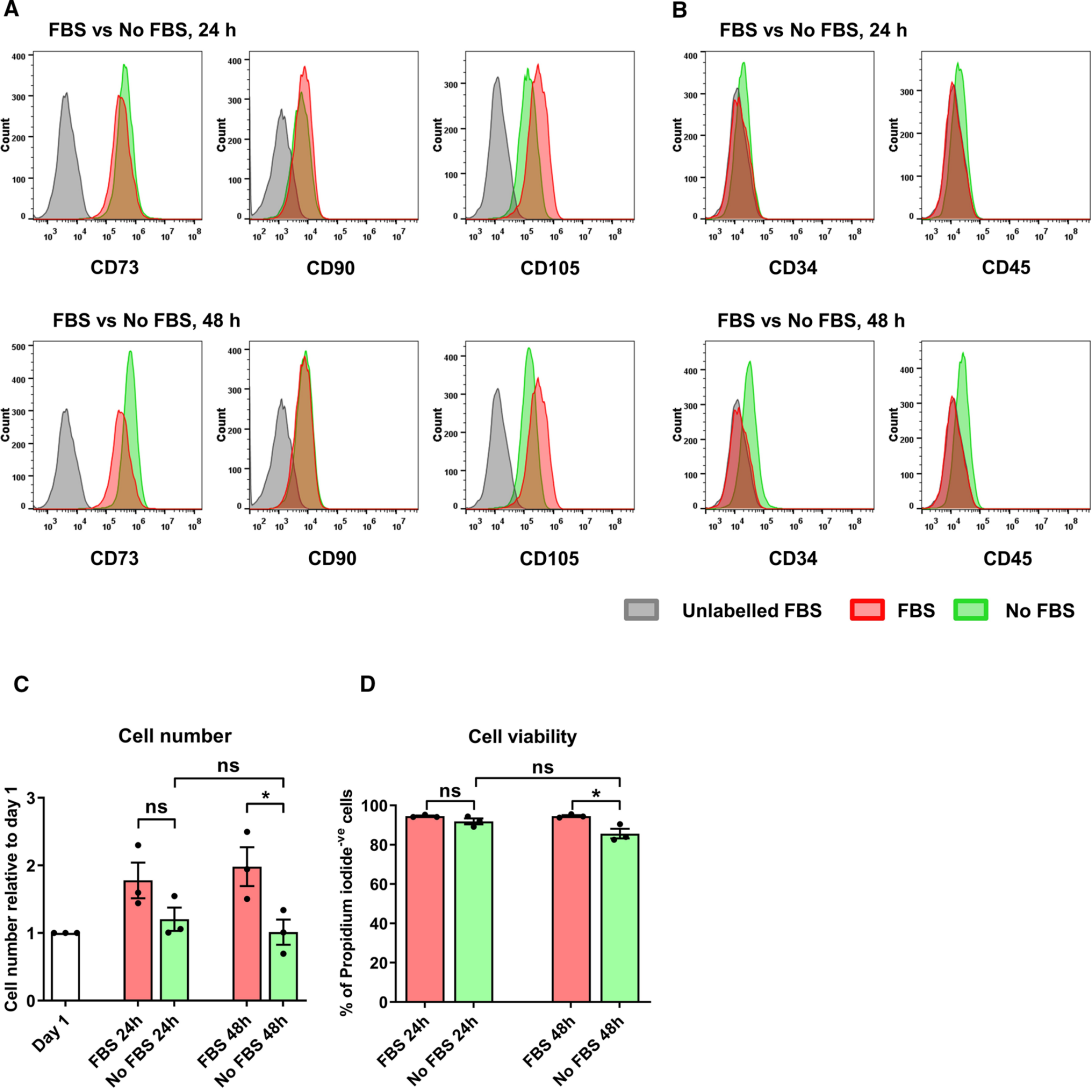
Characterisation of SS-hAFSCs after different incubation timings with or without serum. SS-hAFSCs were incubated for 24 h or 48 h in serum-supplemented or serum-free medium. **a**, **b** Membrane markers present on (**a**) or absent of (**b**) SS-hAFSCs after incubation in serum (FBS)-supplemented and serum-free (No FBS) conditions. **c** Cell number after each incubation timing normalised to day 1. *n* = 3. **d** Live cells (propidium iodide (PI)-negative) after different incubation timings and conditions. *n* = 3. *p < 0.05, *ns* non-significant (*p* > 0.05), one-way repeated-measures ANOVA with Tukey’s post hoc test

Serum-free culture significantly reduces subsequent protein and lipoprotein contamination of the sEV isolates [56]. To confirm that SS-hAFSC retained expression of MSC markers and viability during serum-free culture, cells were incubated for 24 h or 48 h in serum-free medium and MSC marker expression and cell death was evaluated. SS-hAFSCs retained expression of the MSC markers in serum-free conditions with a slight reduction in CD105 expression (Fig. 1a, b) and a mild cell elongation (Supplementary Fig. 2a), both reported previously for MSCs [38]. Despite a cessation of proliferation, cell numbers were maintained even after 48 h of serum-free incubation (Fig. 1c). Flow cytometry showed cell survival of > 85% (Fig. 1d) of which ~ 15% were undergoing early apoptosis (Supplementary Fig. 2b, c). These values were very similar to the previously obtained ones for cell viability of hAFSCs in a serum-free environment [6]. Importantly, despite a slight increase in cell death after a 48-h serum-free compared to serum-supplemented culture, there was no difference in viability between 24 and 48 h serum-free incubation (Fig. 1d and Supplementary Fig. 2b, c).

Overall, 24 h or 48 h serum-free incubation had only subtle effects on SS-hAFSCs’ viability and expression of MSC markers, with negligible changes between 24 and 48 h of serum-free culture.

### Isolation and characterisation of SS-hAFSCs sEVs

**Fig. 2:**
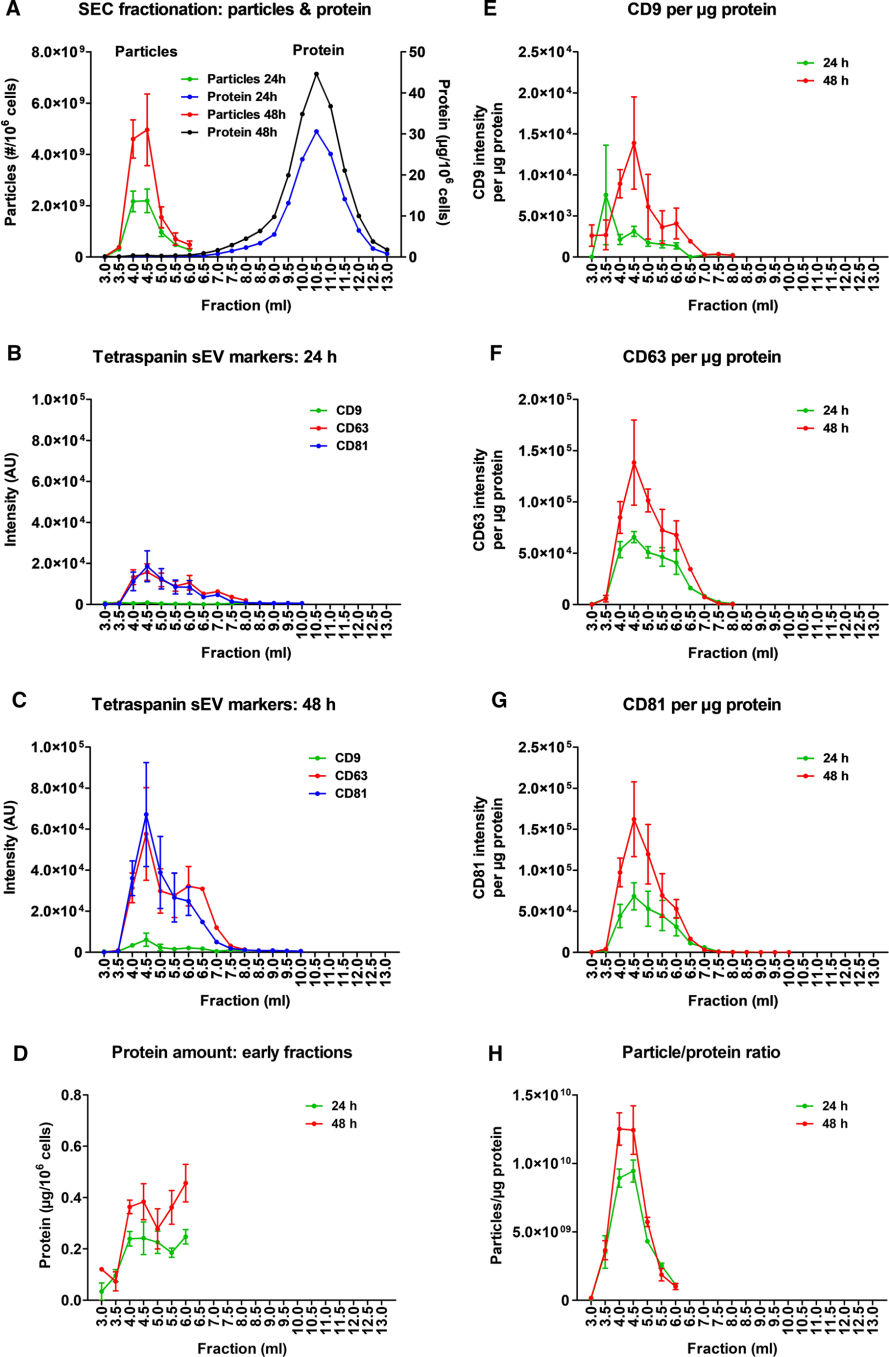
Characterisation of SS-hAFSC sEVs after different incubation timings. sEVs were isolated by SEC of serum-free medium conditioned by SS-AFSCs for 24 h or 48 h. **a** Particle and protein levels of SEC fractions measured by NTA and BCA assays, respectively. *p* < 0.01 for Particles 24 h vs Particles 48 h. **b**, **c** Exosome-specific tetraspanin markers of SEC fractions obtained from 24 h (**b**) or 48 h (**c**) incubation of SS-AFSCs measured by DELFIA. *p* < 0.01 for CD9; *p* < 0.001 for CD63 and CD81. **d** Protein amount of early SEC fractions. Note the low protein quantities and the early protein peak. *p* < 0.001. **e**, g CD9 (**e**), CD63 (**f**), and CD81 (**g**) signal of SEC fractions normalised to protein amount. *p* < 0.05 for **e**; *p* < 0.01 for **g**; *p* < 0.001 for **f**. **h** Particle/protein ratio of early SEC fractions. *p* < 0.05. *n* = 3 where error bars are present and *n* = 1 where absent. Curves compared by two-way repeated-measures ANOVA (points with *n* = 3 included)

To isolate SS-hAFSC sEVs, ultrafiltration combined with SEC of serum-free conditioned medium was performed after 24 h or 48 h of incubation. SEC easily resolved particles (peak elution at 4.0–4.5 ml) from soluble-protein contaminants (peak elution at 10.5 ml) (Fig. 2a). DELFIA confirmed the presence of exosome marker proteins, the tetraspanins CD9, CD63, and CD81, in early SEC fractions with a peak at 4.5 ml (Fig. 2b, c), which coincided with an early protein peak (Fig. 2d). The early SEC fractions contained more particles, protein, and exosome marker levels after 48 h compared to 24 h incubation (Fig. 2a–d). Furthermore, sEV purity was greater after 48 h than 24 h culture, as determined by the ratio of CD9, CD63, or CD81 markers to protein (Fig. 2e–g), and by the ratio of particle-to-protein content (a marker of EV purity [19, 63]) (Fig. 2h).

**Fig. 3:**
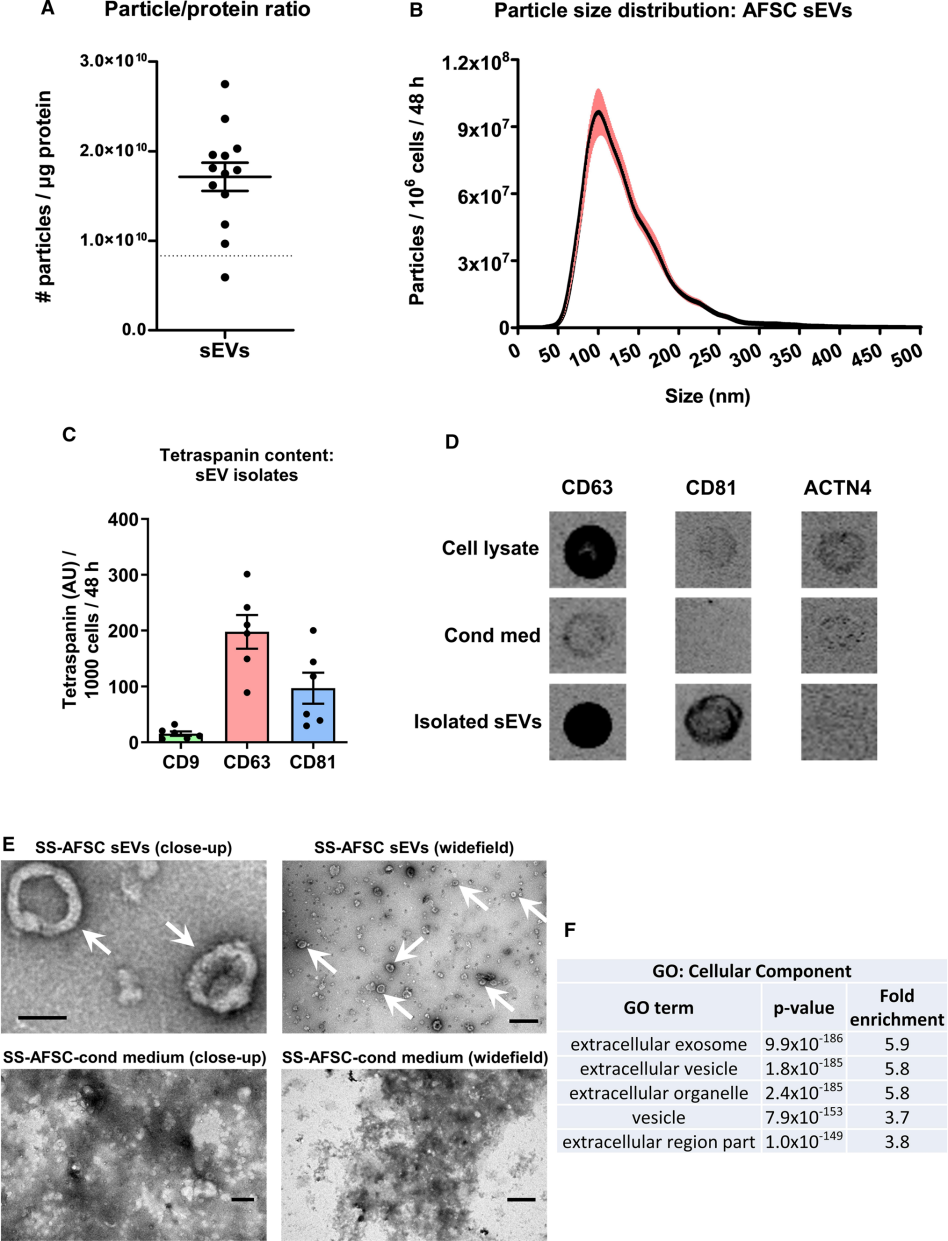
Characterisation of pooled SS-hAFSC sEVs. sEVs were isolated by SEC of serum-free medium conditioned by SS-hAFSCs for 48 h and sEV-rich fractions were pooled and analysed. **a** Particle/protein ratio. *n* = 13. Dotted line–theoretical ratio of 8.3 × 10^9^ particles/µg protein reported for a pure population of sEVs [19]. **b** Particle-size distribution of SS-hAFSC sEVs. *n* = 25. Red lines represent SEM. **c** Levels of tetraspanin exosome-specific markers CD9, CD63, and CD81. *n* = 6. **d** Dot blots for CD63 and CD81 and ACTN4 (alpha-actinin-4—medium/large EV-specific marker). **e** TEM images of SS-hAFSC sEVs and SS-hAFSC-conditioned medium. Arrows point to sEVs. Scales: top left—100 nm; top right—500 nm; bottom left—200 nm; bottom right—1 µm. F: Functional enrichment analysis of the proteins in SS-hAFSC sEVs. The top 5 gene ontology (GO) cellular component terms are shown

Pooled sEV-rich SEC fractions had ~ 99.5% of the soluble protein removed and their ratio of particle-to-protein content reached 1.7 ± 0.2 × 10^10^ particles/µg protein, indicative of a very high purity (Fig. 3a). The majority of SS-hAFSC sEVs exhibited sizes typical for exosomes (30–150 nm) [33, 42], with some medium-sized EVs (Fig. 3b), and they expressed characteristic tetraspanin exosome markers (Fig. 3c). Additionally, there was a dramatic enrichment of CD63 and CD81 in the SS-hAFSC sEV samples compared to conditioned medium, and absence of alpha-actinin-4 (ACTN4), a characteristic protein of medium and large EVs [33], in the isolated sEVs (Fig. 3d). Transmission electron microscopy confirmed the presence of sEVs with characteristic concave disc shapes [52] in the isolates (Fig. 3e) and the absence of impurities which were clearly visible as aggregated dense material in the starting conditioned medium (Fig. 3e). Using LC–MS/MS, SS-hAFSC sEVs were found to be enriched in multiple EV markers [33] including: CD9, CD63, CD81, syntenin-1, TSG101, ALIX, ADAM10, flotillins, annexins, CD47, CD90, NT5E, and CAV1. Furthermore, they were depleted or devoid of non-sEV markers [33] including: HSP90B1, HSPA5, GOLGA2, LMNA, KRT18, and ACTN4. SS-hAFSC sEVs were also highly enriched in proteins associated with the Gene Ontology Cellular Component terms related to extracellular vesicles and exosomes (*p* < 1 × 10^–150^, Fig. 3f, Supplementary Proteomics Table). Notably, bovine apolipoproteins were not detected, implying the absence of carryover of lipoprotein particles to the isolated EVs.

Overall, 48 h serum-free incubation of SS-hAFSCs provided increased yield and purity of the obtained vesicles. To minimise soluble-protein contamination for our functional experiments, we used pooled sEV-rich fractions 4.0 ml, 4.5 ml, and 5.0 ml after 48 h incubation in serum-free medium, hereafter labelled as “SS-hAFSC sEVs”.

### SS-hAFSC sEVs are cardioprotective in vivo, but do not protect isolated cardiomyocytes in vitro

**Fig. 4:**
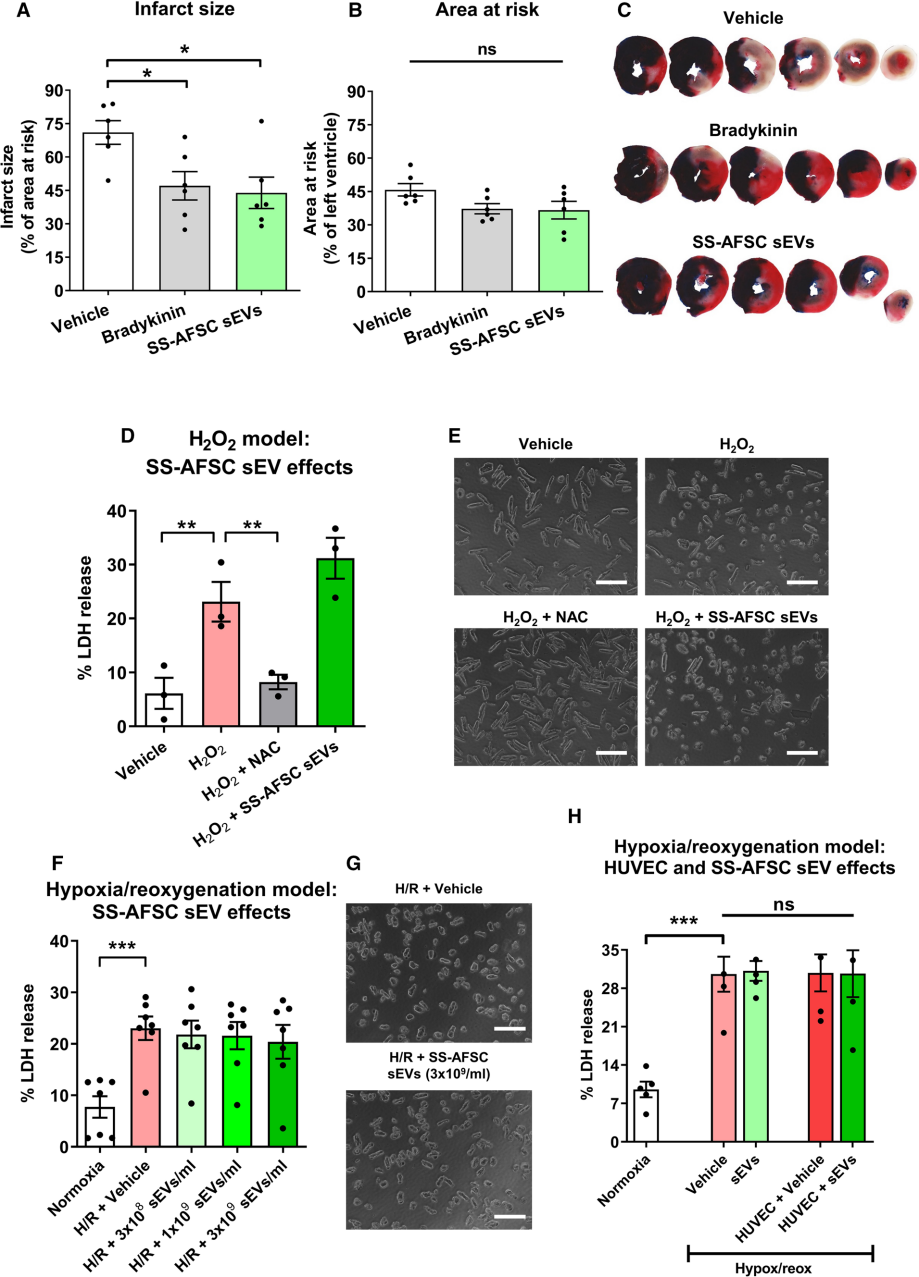
Effects of SS-hAFSC sEVs on cardioprotection in vivo and in vitro*.* **a** Infarct size as a percentage of the area at risk (ischaemic area) in a rat ischaemia–reperfusion injury model. Vehicle (PBS), Bradykinin (40 µg/kg), or SS-hAFSC sEVs (2 × 10^11^ particles/rat) were administered intravenously, 2 min prior to reperfusion. *n* = 6. *p < 0.05, one-way ANOVA with Dunnett’s post hoc test. **b** Area at risk as a percentage of left ventricle area. *n* = 6. *p* > 0.05, one-way ANOVA. **c** Representative images of **a** and **b**. **d**, **e** H_2_O_2_-induced death of primary cardiomyocytes treated with 40 µM H_2_O_2_ and Vehicle (water, PBS), *N*-acetyl-l-cysteine (NAC, 300 µM), or SS-hAFSC sEVs (1 × 10^10^ particles/ml). Cell death was assessed by LDH release. **p < 0.01, one-way repeated-measures ANOVA with Dunnett’s post hoc test. *n* = 3. Representative images are shown on **e**. Scale: 200 µm. **f**, **g** Hypoxia/reoxygenation-induced death of primary cardiomyocytes in the absence or presence of SS-hAFSC sEVs. Cell death was assessed by LDH release. ***p < 0.001, one-way repeated-measures ANOVA with Dunnett’s post hoc test. *n* = 7. Representative images are shown on **g**. Scale: 200 µM. **h** Effects of the stable sEV-treated HUVEC secretome in the hypoxia/reoxygenation-induced cardiomyocyte death model. Primary cardiomyocytes were treated with non-conditioned (Vehicle or sEVs at 1 × 10^10^ sEVs/ml) or HUVEC-conditioned medium (vehicle-treated: HUVEC + Vehicle, or sEV-treated: HUVEC + sEVs at 1 × 10^10^ sEVs/ml). ***p < 0.001, *ns* non-significant, one-way repeated-measures ANOVA with Tukey’s post hoc test. *n* = 5

To test the hypothesis that SS-hAFSC sEVs can protect the myocardium in vivo, we used a rat model of 30-min myocardial ischaemia followed by 2-h reperfusion with intravenous treatment administration 2 min prior to reperfusion. As expected, a positive control (bradykinin) reduced infarct size (Fig. 4a). SS-hAFSC sEVs were also found to be cardioprotective, decreasing infarct size by 27%, from 71 ± 5% to 44 ± 7% (Fig. 4a, c). The ischaemic area (i.e., area at risk) was not different between the groups (Fig. 4b, c).

Therefore, to investigate the potential cardioprotective mechanism of the SS-hAFSC sEVs, we used a model of reactive oxygen species (ROS)-induced death of primary adult rat ventricular cardiomyocytes. Initial experiments established a model, showing that H_2_O_2_ induces cardiomyocyte death with an EC_50_ of 41 ± 4 µM (Supplementary Fig. 4a) and *N*-acetyl-l-cysteine (NAC) can efficiently protect against H_2_O_2_-induced death with the most consistent response conferred by 300 µM NAC (Supplementary Fig. 4b, c). However, no protective effect of SS-hAFSC sEVs was seen in this assay (Fig. 4d, e).

Next, to investigate the cardioprotective mechanism in a more physiological model, we subjected cardiomyocytes to hypoxia/reoxygenation in vitro. Hypoxia/reoxygenation significantly increased cell death in comparison to the control normoxic conditions, but none of the doses of SS-hAFSC sEVs had protective effects in this model (Fig. 4f, g). This led us to hypothesize that the cardioprotection seen in vivo may be via an indirect mechanism via signalling from the endothelium. To test this, conditioned medium from HUVECs treated in vitro with SS-hAFSC sEVs or vehicle was collected, concentrated, and used to treat primary cardiomyocytes prior to hypoxia/reoxygenation. Similar to our previous observations, administration of SS-hAFSC sEVs alone did not protect cardiomyocytes. Furthermore, no protection was seen with conditioned medium from sEV-treated HUVECs (Fig. 4h), indicating that the stable secretome of sEV-treated endothelial cells does not mediate the cardioprotective effects of SS-hAFSC sEVs.

### SS-hAFSC sEVs promote migration of endothelial cells, but are not proangiogenic in vitro

To assess the angiogenic potential of the isolated SS-hAFSC sEVs, HUVEC migration, proliferation, and tube formation in response to sEVs were studied.

**Fig. 5:**
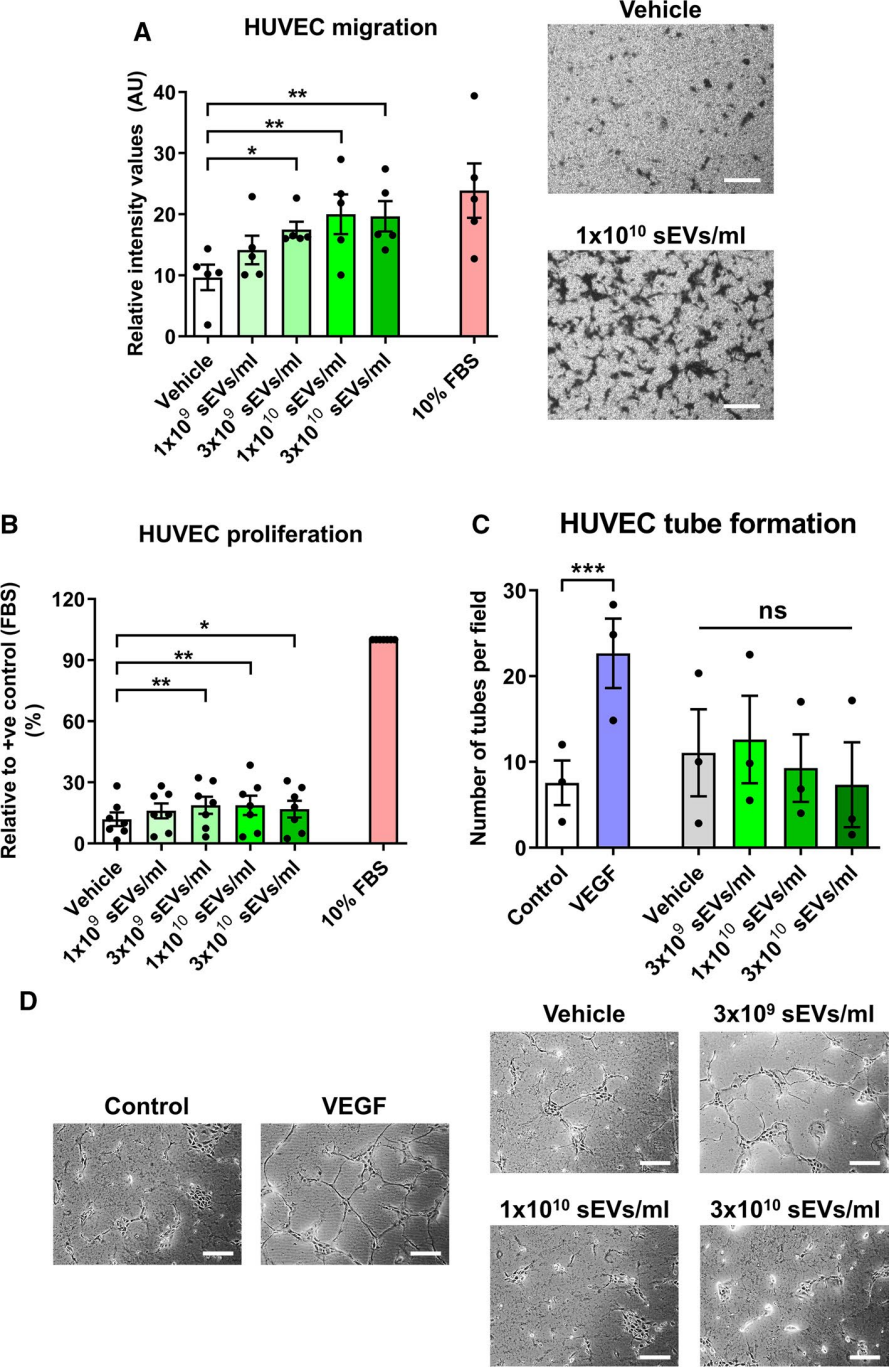
Effects of SS-hAFSC sEVs on angiogenesis in vitro. **a** Using a modified Boyden’s Chamber assay, HUVEC migration was assessed in response to vehicle (PBS), SS-hAFSC sEVs or 10% FBS (control). Migration is presented as mean staining intensity. *p < 0.05, **p < 0.01, one-way repeated-measures ANOVA with Dunnett’s post hoc test. *n* = 5. Right panels—representative images (as indicated). Scale: 200 µm. **b** HUVEC proliferation was assessed using an MTT assay in the presence of vehicle (PBS) or SS-hAFSC sEVs. Proliferation is presented relative to a positive control of 10% FBS. *p < 0.05, **p < 0.01, one-way repeated-measures ANOVA with Dunnett’s post hoc test. *n* = 7. **c**, **d** In vitro angiogenesis was assessed using an HUVEC tube formation assay in the absence of treatments (Control) or in the presence of VEGF (25 ng/ml), vehicle (PBS) or SS-hAFSC sEVs. Results are presented in **c** as number of tubes per field of analysis. ***p < 0.001, *ns* non-significant, one-way repeated-measures ANOVA with Tukey’s post hoc test. *n* = 3. Representative images are shown in **d**. Scale: 200 µm

SS-hAFSC sEVs promoted migration of endothelial cells in a dose-dependent manner with concentrations ≥ 3 × 10^9^ particles/ml being effective (Fig. 5a). sEV promigratory potential was marked and reached levels similar to the serum positive control used (Fig. 5a). Intriguingly, SS-hAFSC sEVs promoted endothelial cell proliferation, but these effects were subtle compared to the baseline proliferation levels and the serum control (Fig. 5b). Additionally, none of the doses of SS-hAFSC sEVs stimulated tube formation of endothelial cells in vitro (Fig. 5c, d).

Overall, SS-hAFSC sEVs had minor effects on proliferation and tube formation of endothelial cells, but potently stimulated endothelial cell migration in vitro.

### SS-hAFSC sEVs carry promigratory factors

To obtain a profile of potential promigratory and proangiogenic factors present in the SS-hAFSC sEVs that may be responsible for the observed promigratory effects, two different strategies were used: protein arrays and proteomic analysis.

**Fig. 6:**
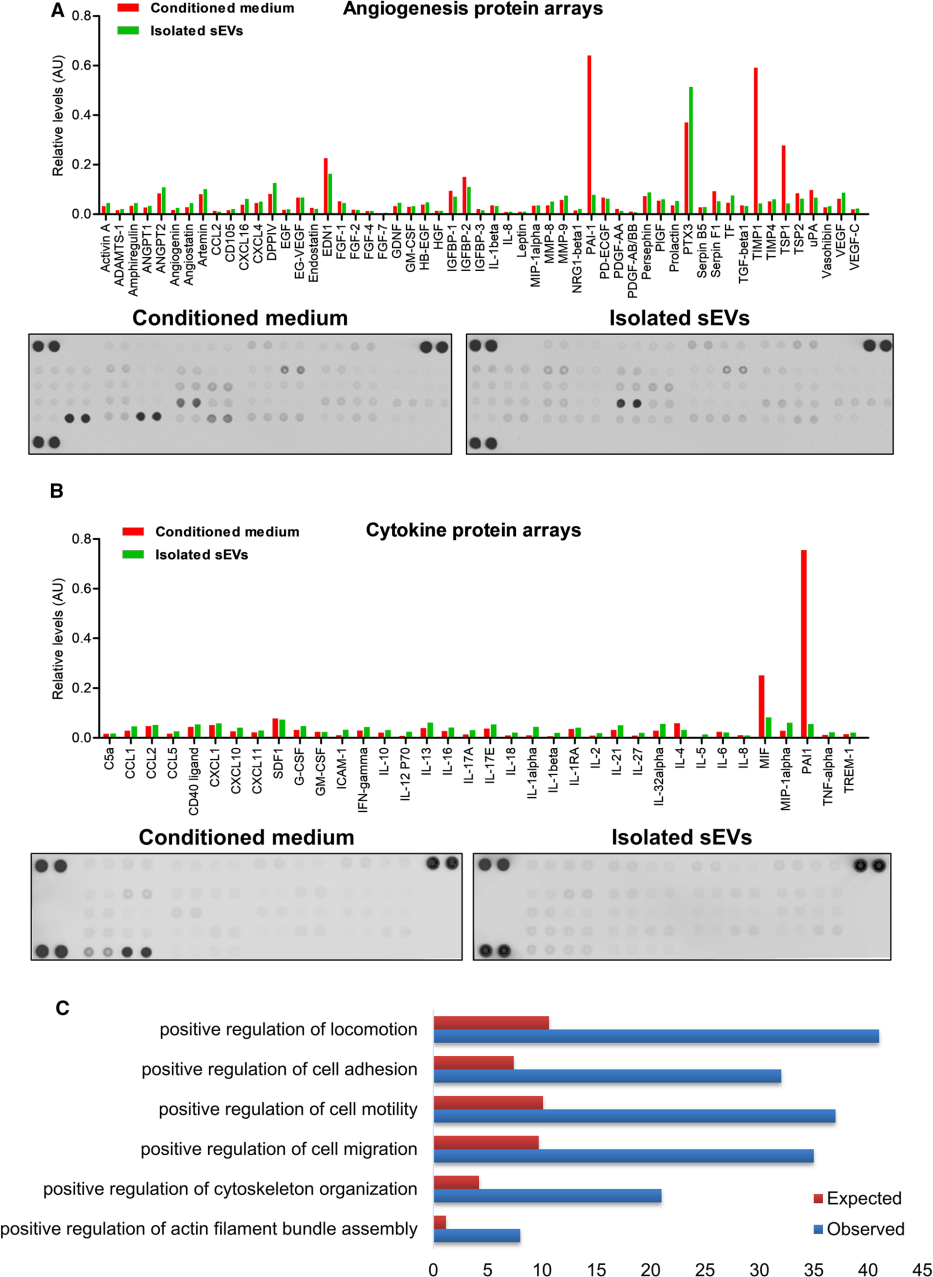
SS-hAFSC sEVs cargo. **a**, **b** Protein arrays for detection of angiogenic factors (**a**) and cytokines (**b**) in SS-hAFSC-conditioned medium and SS-hAFSC sEVs. Representative images of the membranes also shown. **c** Gene ontology (GO): Biological Process terms associated with cell migration and overrepresented in SS-hAFSC sEVs. Proteomic analysis of SS-hAFSC sEV-enriched proteins (> 1.5 times) and SS-hAFSC sEV-exclusive proteins was performed. Red bars—expected number of proteins (based on the number of proteins in the human proteome); blue bars—observed number of proteins. See Supplementary Proteomics Table for full results

Using angiogenesis and cytokine protein arrays, multiple potential proangiogenic factors were found to be present in the conditioned medium and the sEV isolates (Fig. 6a, b). Some factors, such as PTX3, were clearly enriched in the isolated SS-hAFSC sEV samples, while others, such as PAI1, TIMP1, and TSP1, were mostly found in the conditioned medium (Fig. 6a, b). When examining protein expression using a cytokine array, many of the cytokines were identified, although none were present at particularly high levels in the sEVs. The most abundant ones in the sEVs were MIF and SDF1 (Fig. 6b).

Proteomic analysis confirmed most of the observations from the protein arrays (Supplementary Proteomics Table). This approach identified further potential promigratory mediators in the SS-hAFSC sEV samples such as BGN [29] and RTN4 [1] which were enriched in the sEV samples compared to the starting conditioned medium.

Next, we focussed on the sEV-exclusive and sEV-enriched (> 1.5 times) proteins. Gene Ontology classification according to Biological Process yielded numerous terms overrepresented in the sEV proteome including those associated with cell migration (Fig. 6c). Interestingly, proteins associated with the term “positive regulation of locomotion” did not cluster together on a protein network (Supplementary Fig. 5 and Supplementary Proteomics Table), indirectly suggesting that the sEVs may stimulate migration via multiple independent mechanisms.

A recent study systematically compared the angiogenic potential of the secretome from several types of human MSCs including foetal Wharton’s jelly MSCs, and found that biological processes related to angiogenesis were enriched in the secretomes of all MSC types, with the most complete angiogenic profile detected in the foetal MSC secretome [30]. Our proteomic analysis identified 106 additional, unique proteins in the SS-AFSC sEVs that were not detected in any of the adult or foetal MSC secretomes in this study (Supplementary Proteomics Comparison table). Interestingly, gene ontology analysis of these 106 proteins showed that the unique proteins from SS-AFSC sEVs associate with terms related to angiogenesis and (cardio) vascular development (Supplementary Proteomics Comparison table). Therefore, it will be interesting to compare the proangiogenic capacity of SS-AFSC with other types of MSCs in future studies.

Overall, SS-hAFSC sEVs carried diverse proteins, some of which are likely to be associated with the promigratory effects observed here. Intriguingly, some of the most abundant proteins, including SDF1 [13], MIF [31], PTX3 [32], RTN4 [1], and BGN [29], have previously been shown to exhibit chemotactic activities outside of EVs.

### SS-hAFSC sEVs are the active promigratory components of SS-hAFSC-conditioned medium

**Fig. 7:**
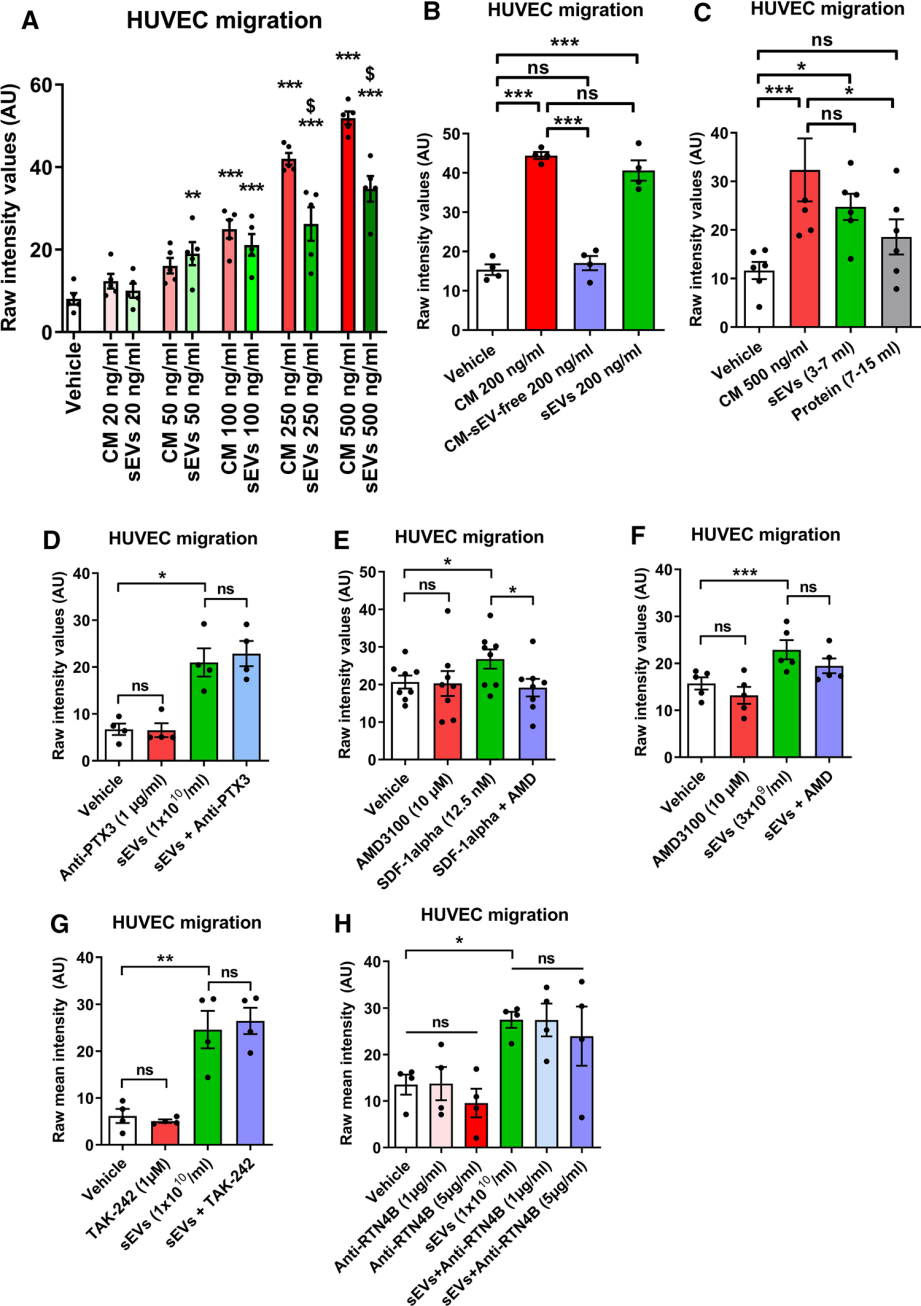
Comparison of the promigratory effects of SS-hAFSC-conditioned medium and isolated SS-hAFSC sEVs and the mediators of these effects in the vesicular cargo. **a** HUVEC migration in response to SS-hAFSC-conditioned medium (CM) or isolated SS-hAFSC sEVs. **p < 0.01 vs Vehicle, ***p < 0.001 vs Vehicle, $ *p* < 0.001 vs CM (respective dose), one-way repeated-measures ANOVA with Tukey’s post hoc test. *n* = 5. **b** HUVEC migration in response to SS-hAFSC-conditioned medium (CM), SS-hAFSC-conditioned medium depleted of sEVs (CM-sEV-free) or isolated SS-hAFSC sEVs. ***p < 0.001, *ns* non-significant, one-way repeated-measures ANOVA with Dunnett’s post hoc test. *n* = 4. **c** HUVEC migration in response to SS-hAFSC-conditioned medium (CM) or SEC fractions of CM: sEVs (3–7 ml) or Protein (7–15 ml). sEVs and protein were normalised to the CM volume. *p < 0.05, ***p < 0.001, one-way repeated-measures ANOVA with Tukey’s post hoc test. *n* = 6. **d**–**h** HUVEC migration in response to SS-hAFSC sEVs in combination with various inhibitors or antibodies: Anti-PTX3 antibody (**d**), *n* = 4; AMD3100—CXCR4 inhibitor (**e** and **f**). *n* = 8 (**e**). *n* = 5 (**f**). TAK-242—TLR4 inhibitor (**g**). *n* = 4. Anti-RTN4B antibody (**h**). *n* = 4. *p < 0.05, **p < 0.01, ***p < 0.001, *ns* non-significant (*p* > 0.05), one-way repeated-measures ANOVA with Tukey’s post hoc test. Migration is presented as mean staining intensity in all panels

Similar to the isolated SS-hAFSC sEVs, SS-hAFSC-conditioned medium demonstrated dose-dependent promigratory effects on endothelial cells (Fig. 7a). Intriguingly, the promigratory effects of the conditioned medium were completely lost when it was depleted of sEVs using our isolation protocol, indicating that the vesicles are the active chemotactic mediator of the SS-hAFSC secretome (Fig. 7b). It is possible, however, that this experiment was confounded by the multiple ultracentrifugation and ultrafiltration steps involved. Therefore, in a further experiment, we directly compared the sEV-rich fractions (3.5 ml–7.0 ml) to sEV-poor (i.e., soluble protein-rich) fractions (7.0 ml–15.0 ml) of the SS-hAFSC-conditioned medium, omitting the ultrafiltration step. This experiment confirmed that the soluble-protein fraction of the conditioned medium has no significant promigratory effect, in comparison to the sEV fraction, which completely recapitulated the effects of the conditioned medium (Fig. 7c).

To investigate the mechanism of sEV-induced endothelial cell migration, we used pathway inhibitors of some abundant, candidate factors identified by protein arrays or proteomic analysis. Application of a neutralising anti-PTX3 antibody had no effect on sEV-induced migration (Fig. 7d). A CXCR4 inhibitor efficiently blocked migration in response to recombinant SDF1α (Fig. 7e), but did not significantly reduce sEV-induced endothelial chemotaxis (Fig. 7f). Similarly, an inhibitor of TLR4 had no effect on endothelial cell migration stimulated by SS-hAFSC sEVs (Fig. 7g). Finally, application of a neutralising antibody against RTN4B also had no effect on the sEV-induced endothelial cell chemotaxis (Fig. 7h).

In summary, despite the complexity of the soluble-protein secretome of SS-hAFSCs, sEVs were found to be the active promigratory factor of the SS-hAFSC-conditioned medium. Furthermore, some of the abundant factors present in the sEVs (PTX3, SDF1, MIF, BGN, and RTN4B) were excluded as potential mediators of the promigratory effects.

### SS-hAFSC sEV-induced migration requires PI3K signalling

**Fig. 8:**
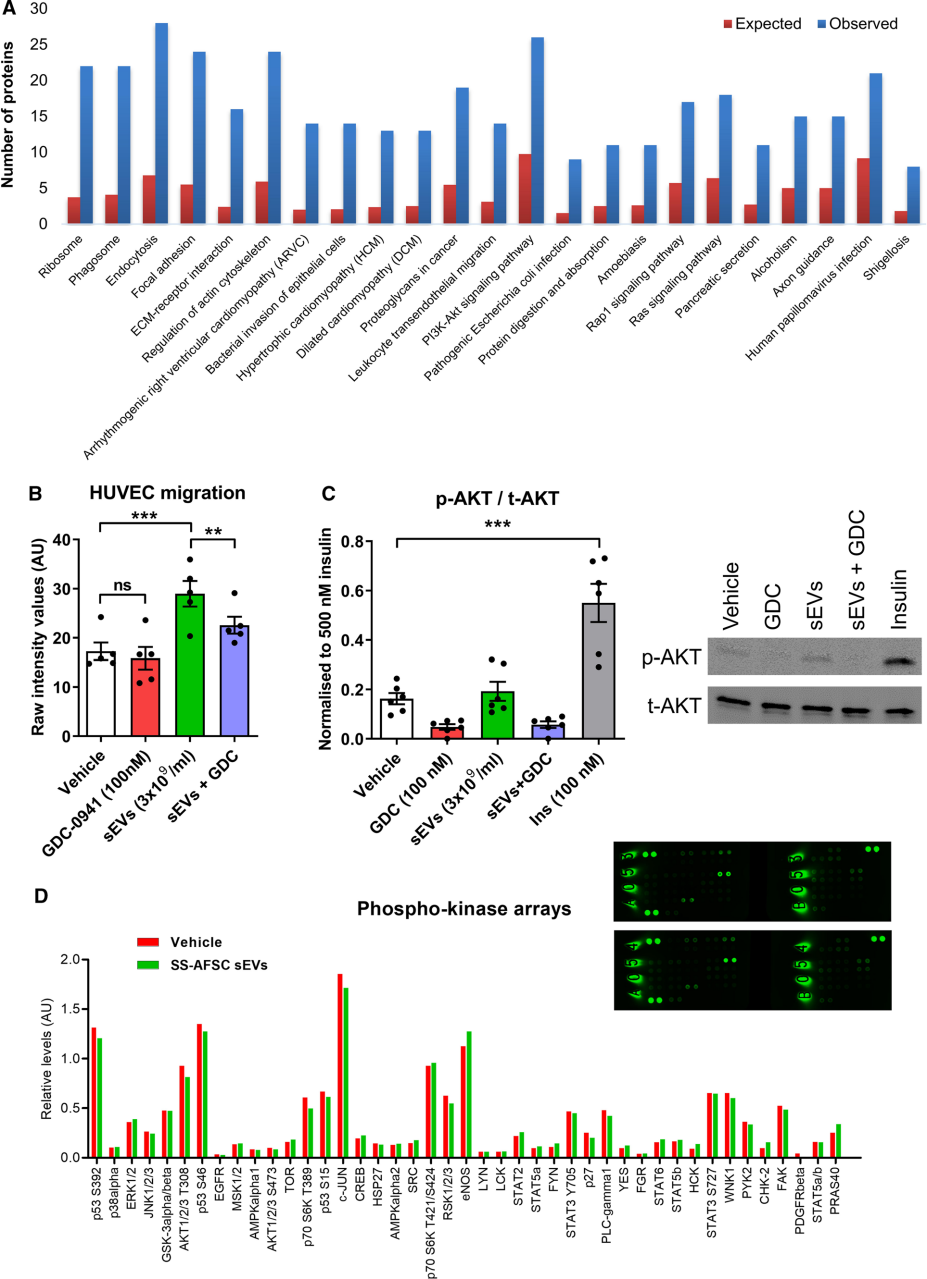
Mechanism of SS-hAFSC sEV-induced endothelial cell migration. **a** Proteomic analysis of SS-hAFSC sEV-enriched proteins (> 1.5 times) and SS-hAFSC sEV-exclusive proteins was performed. Overrepresented KEGG pathways are shown ranked by their *p* values for enrichment. Red bars—expected number of proteins (based on the number of proteins in the human proteome); blue bars—observed number of proteins. **b** HUVEC migration in response to SS-hAFSC sEVs and vehicle (PBS, DMSO) or PI3K pathway inhibitor (GDC-0941). **p < 0.01, ***p < 0.001, *ns* non-significant (*p* > 0.05), one-way repeated-measures ANOVA with Tukey’s post hoc test. *n* = 5. **c** Western blotting results for total and phosphorylated AKT in endothelial cells. HUVECs were incubated with Vehicle (PBS, DMSO), GDC-0941, SS-hAFSC sEVs, SS-hAFSC sEVs + GDC-0941 or insulin (control) for 15 min. Results are presented relative to 500 nM insulin control. ***p < 0.001, one-way repeated-measures ANOVA with Tukey’s post hoc test. *n* = 6. Representative images shown on the right. p-AKT: phosphorylated AKT, t-AKT: total AKT. **d** Phospho-kinase array for detection of phosphorylated kinases/kinase targets in endothelial cells. HUVECs were incubated with vehicle (PBS) or SS-hAFSC sEVs (1 × 10^10^ particles/ml) for 15 min. Results are presented relative to control proteins. Images of the membranes shown in the top right corner: Vehicle (top, A053 and B053) and SS-hAFSC sEVs (bottom, A054 and B054)

Mechanistically, the PI3K pathway is a known player in endothelial cell migration [51]. Additionally, KEGG pathway analysis showed a significant overrepresentation of proteins associated with the PI3K-AKT signalling pathway in SS-hAFSC sEVs (Fig. 8a and Supplementary Proteomics Table). Intriguingly, inhibiting the PI3K pathway in endothelial cells reduced sEV-stimulated migration by 54 ± 15% (Fig. 8b). However, administration of sEVs led to only a subtle, non-significant increase in phosphorylation of AKT, a downstream PI3K target (Fig. 8c). We observed the same non-significant increase when using higher sEV concentration (1 × 10^10^ particles/ml) or additional downstream PI3K targets (i.e., PRAS40) (Supplementary Fig. 5a, b). Furthermore, a phospho-kinase array showed no increase in phosphorylation of 43 different signalling kinases or kinase targets in the endothelial cells for both short-term (i.e., 15 min; Fig. 8d) or long-term (i.e. 3 h; Supplementary Fig. 5c) incubation with sEVs.

In summary, SS-hAFSC sEV-promoted endothelial cell migration required, but was not solely dependent on, PI3K signalling in the target endothelial cells.

## Discussion

In this study, we used SEC to isolate highly pure, foetal MSC sEVs from medium conditioned by SS-hAFSCs. Our study provides the most extensive characterisation of the harvested AFSC sEVs to date and shows that they bear cardioprotective and marked promigratory abilities. These findings unveil the potential for foetal AFSC sEVs to be used as a new therapeutic in the setting of myocardial infarction.

The search for a perfect isolation technique to obtain completely pure sEVs from biological fluids or conditioned cell-culture medium continues. There is a considerable body of evidence that SEC may be among the best methods to purify vesicles from contaminants of a sample without compromising their integrity [8, 37, 64]. However, we [55, 56] and others [53] have previously shown that SEC is highly prone to co-isolate soluble proteins and lipoproteins with sEVs from samples containing blood products such as serum. Hence, in this report, we used serum-free medium for SS-hAFSC culture to eliminate confounding contaminants and exploit the full potential of SEC to isolate sEVs with a better purity and improved functional efficacy [39]. Importantly, the SS-hAFSCs cultured under serum-free conditions showed only minor alterations in cell viability, morphology and expression of MSC markers, and longer, 48-h incubation timings yielded significantly more sEVs with a better purity than a 24-h incubation.

We demonstrate for the first time that SS-hAFSC sEVs can protect the animal heart from ischaemia–reperfusion injury when using a clinically suitable administration technique and injection timing (i.e., intravenous injection just prior to myocardial reperfusion). While the previous findings indicated that sEVs isolated from adult multipotent progenitors may have benefits in the setting of myocardial infarction, their clinical applicability is less clear because of ethical concerns (e.g., using embryonic stem cells to derive MSCs [34]) or difficulty of the process for obtaining the cells (e.g., differentiation of embryonic stem cells [34] and cardiac biopsies from diseased patients [7, 15]). Our findings are especially relevant in light of recent reports, demonstrating that sEVs from MSCs [45] or cardiosphere-derived cells [25] cannot exert cardioprotective benefits if delivered to the bloodstream. Since intravenous administration of MSCs or MSC-conditioned medium [59] is cardioprotective, it could be speculated that the intravenous cardioprotection of SS-hAFSC sEVs observed here may be due to their potent foetal nature. In support of the argument that SS-hAFSC sEVs are exceptionally potent, the dose of sEVs which we administered to rats in terms of protein content was ~ 40–70 µg/kg and there is only one previous study of MSC sEVs which used a dose lower than this one [3].

In the current study, we investigated, for the first time, the effects of AFSC sEVs in a hypoxia/reoxygenation-induced primary cardiomyocyte death model. However, SS-hAFSC sEVs did not protect primary cardiomyocytes despite their potential to reduce infarct size in vivo. The lack of cytoprotective effects was also confirmed using a ROS-induced cell death model. Cardioprotection may also be mediated via an indirect mechanism, e.g., through effects on the endothelial cells [18]. Interestingly, no pro-survival effects of the secretome of HUVECs pretreated with SS-hAFSC sEVs were observed here. It should be noted that our model only assayed the stable cardioprotectant molecules released by the endothelial cells, and it is possible that released small, unstable molecules (e.g., nitric oxide) are responsible for the in vivo cardioprotection. It is also plausible that SS-hAFSC sEV protective activity is mediated via effects on other organs or cells (e.g., immune cells [15]).

There is some emerging evidence for a role of AFSC sEVs in angiogenesis, but it is not well defined mechanistically [5, 6]. In fact, studies by Balbi et al*.* indicated that bulk AFSCs (not selected for SS-hAFSC) sEVs are not potent stimulators of angiogenesis [5, 6]. Our findings support the previous observations of Balbi et al*. *in vivo [5]. We have shown that SS-hAFSC sEVs promote migration of endothelial cells, but do not considerably affect their proliferation and tube formation. Intriguingly, medium conditioned by SS-hAFSCs was previously shown to promote migration, proliferation, and tube formation of endothelial colony forming cells derived from umbilical cord, but the most pronounced effects were seen in terms of promigratory activity [48].

Using two different approaches, it was demonstrated here that sEVs carry the chemotactic activity of SS-hAFSC-conditioned medium. Given that Balbi et al*.* argued that the presence of both soluble secretome and released sEVs is required to achieve efficient angiogenesis, it is possible that the sEVs are the active promigratory component of the SS-hAFSC-conditioned medium, while the soluble SS-hAFSC secretome may be required for the full angiogenic process (e.g., proliferation and tube formation). This is currently unknown and remains to be investigated.

The finding that SS-hAFSC sEVs are very efficient in promoting migration of endothelial cells but only modestly affect proliferation and do not stimulate tube formation is intriguing and quite unusual. It can be speculated that SS-hAFSC sEVs may only act on part of the angiogenic process. For instance, they may have promigratory but no mitogenic activity similar to other chemotactic factors [21].

Guided by data obtained using protein arrays and proteomics, we have attempted to determine the mediator of the promigratory effects of the SS-hAFSC sEVs. Among the identified putative promigratory proteins, SDF1 is a well-known recruiting factor for endogenous progenitor cells [44, 68] and it induces an angiogenic phenotype of endothelial progenitor cells [66]. Furthermore, SDF1 was previously shown to deliver endothelial-mediated cardioprotection [12] and to provide proangiogenic support to ischaemic hearts decreasing scars and increasing capillary density in vivo [50]. MIF, identified by our protein arrays, also induces chemotaxis via actions on CXCR4 receptor as shown using primary murine lymphocytes [31]. However, in the current study, pharmacological inhibition of CXCR4 did not affect SS-AFSC sEV-induced HUVEC migration, suggesting that vesicular SDF1 and MIF do not mediate this effect.

PTX3 was found to be relatively abundant in the sEV samples by both methods used here, and it showed an enrichment in the sEV isolates compared to the starting conditioned medium. Furthermore, PTX3 has previously been shown to induce the migration of pancreatic cancer cells [32]. However, inhibition of the PTX3 with the use of an antibody did not have an impact on the sEV-promoted endothelial cell migration.

Finally, the lack of effect of the selective TLR4 inhibitor TAK-242 on SS-hAFSC sEV-induced endothelial cell migration eliminated the possibility of TLR4 ligand-driven effects, such as BGN-induced migration [29], or contaminant endotoxin-driven migration [49]. Therefore, we were unable to identify a single protein involved in sEV-promoted endothelial cell migration. Notably, a multitargeted promigratory effect of the sEVs is also conceivable, since proteins associated with positive regulation of cell migration were part of different pathways as seen on our protein interaction network.

Despite our data-driven approach to select potential candidates for promigratory mediators, some of the most abundant proteins identified by LC–MS/MS were only a small fraction of the peptide area of the total proteins identified by label-free quantification. For instance, RTN4 was < 2% of the total peptide area despite being the ninth most abundant protein in the sEV isolates. Interestingly, type I collagens (COL1A1 and COL1A2) represented > 50% of the total peptide area detected indicative of a remarkably high abundance. It remains unknown whether these proteins contribute to HUVEC chemotaxis seen here, but it has been shown that collagen type I can induce morphological changes in endothelial cells and formation of capillary-like structures [65].

Importantly, it cannot be excluded that other SS-hAFSC sEV cargo such as microRNAs [60] or lipids [47] is responsible for their chemotactic effects, although these would have to act over a relatively short time-scale, since the assay only lasted 6 h. It should also be noted that several arguments have been raised against the possibility of miRNA-mediated effects of MSC sEVs, including the low miRNA concentration within EVs, low proportion of miRNA relative to other ribonucleic acid fragments, and the absence of RNA-induced silencing complex (RISC) proteins in the sEVs which are necessary for mature miRNA function [61].

Here, we demonstrated that PI3K signalling in endothelial cells is required for SS-hAFSC sEV-induced migration, although sEVs did not directly activate PI3K in HUVECs. This indicates that basal activity of PI3K pathway is necessary for the directional migration of HUVECs towards a gradient of SS-hAFSC sEVs and compromising the integrity of the endothelial PI3K can reduce the promigratory effects of sEVs. Further to that, administration of sEVs did not lead to an increase of phosphorylation in any of the kinases/kinase targets in the endothelial cells in the panel which we used, so it is likely that the sEVs act via other signalling pathways that have not been investigated here. These observations may be due to a more complex signalling owing to the multifaceted actions of the sEVs.

In conclusion, we established a method to isolate highly pure sEVs from medium conditioned by SS-hAFSCs. SS-hAFSC sEVs were cardioprotective and promigratory, and they were fully responsible for the chemotactic effects of the SS-hAFSC-conditioned medium. Overall, this report sheds light on the cardiovascular effects of sEVs obtained from young, foetal MSCs and may be a basis for future development of therapies for patients who have suffered a myocardial infarction. In future, it will also be interesting to assess the potential benefit of these sEVs in other disease indications affecting the heart including kidney disease [67].

## Electronic supplementary material

Below is the link to the electronic supplementary material.

Supplementary file1 (XLSX 542 kb)

Supplementary file2 (XLSX 124 kb)

Supplementary file3 (DOCX 3333 kb)
