# Supplementary material for: Small extracellular vesicles secreted from human amniotic fluid mesenchymal stromal cells possess cardioprotective and promigratory potential
Source: Basic Res Cardiol. 2020 Mar 7;115(3):26. doi: 10.1007/s00395-020-0785-3 (PMC7060967; doi:10.1007/s00395-020-0785-3)
Supplement: Supplementary file 3 — Supplementary file3 (DOCX 3333 kb) [file 395_2020_785_MOESM3_ESM.docx]

**Supplementary Data**

**Small extracellular vesicles secreted from human amniotic fluid mesenchymal stromal cells possess cardioprotective and promigratory potential**

Kaloyan Takov^1^, Zhenhe He^1^, Harvey Johnston^2^, John Timms^2^, Pascale Guillot^2^, Derek M Yellon^1^, Sean M Davidson^1,*^

^1^ The Hatter Cardiovascular Institute, University College London, London, UK

^2^ EGA Institute for Women’s Health, University College London, London, UK

*Corresponding author: Prof Sean Davidson; The Hatter Cardiovascular Institute, University College London, 67 Chenies Mews, London WC1E 6HX, United Kingdom; Phone: +44 (0)203 447 9894; Email: s.davidson@ucl.ac.uk

# Supplementary Figures

**Supplementary Figure 1**


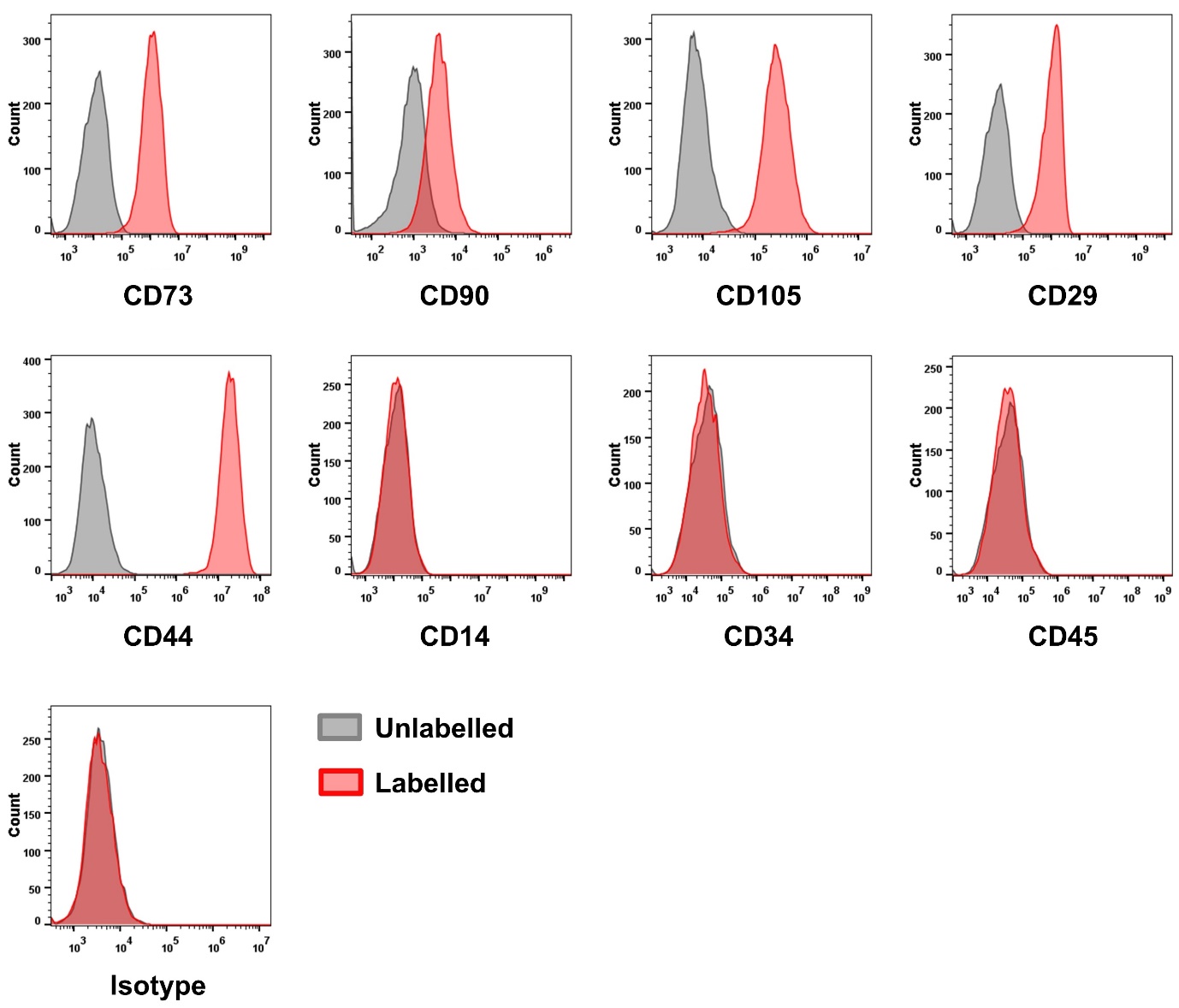


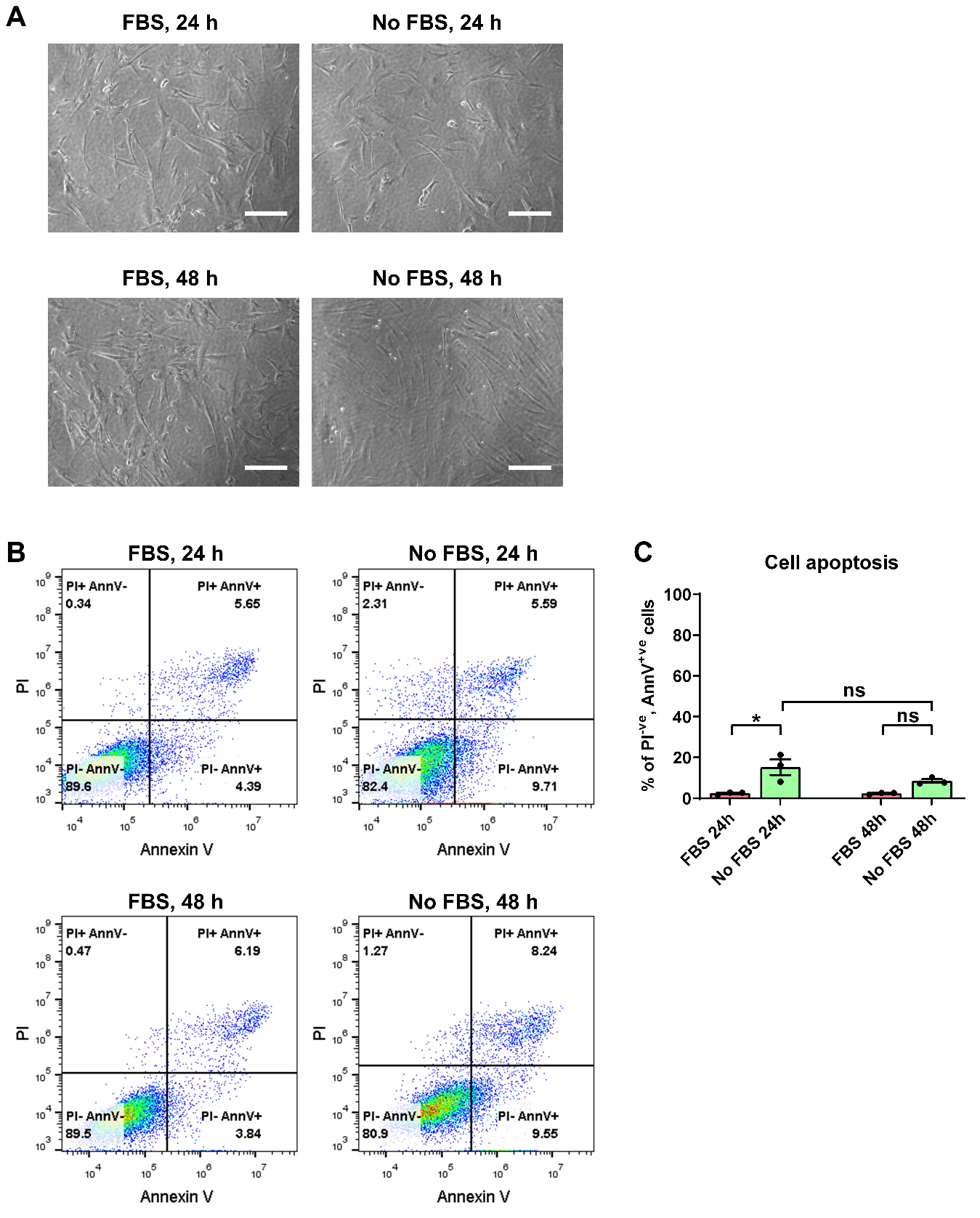


**Supplementary Figure 2**


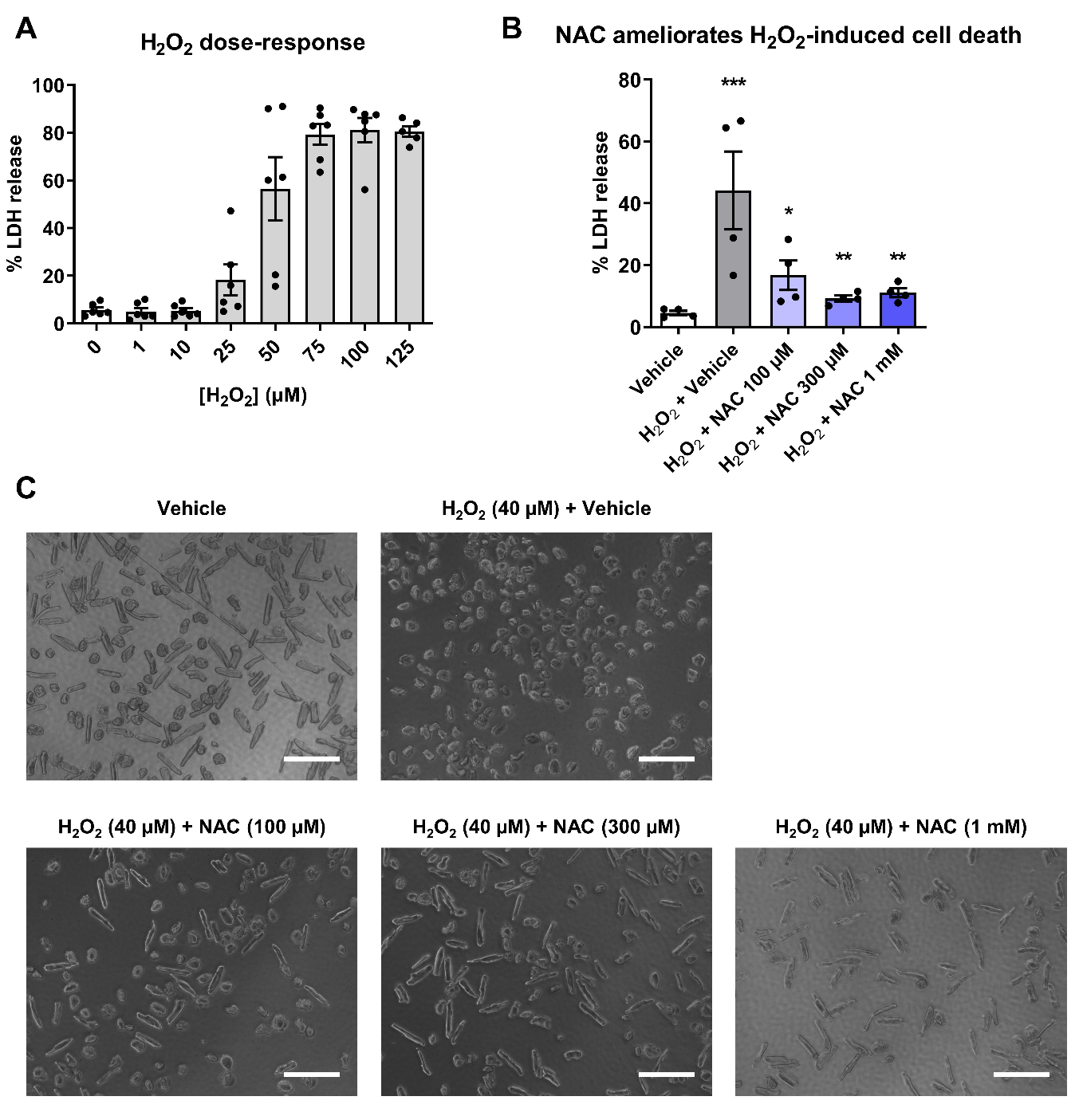


**Supplementary Figure 3**


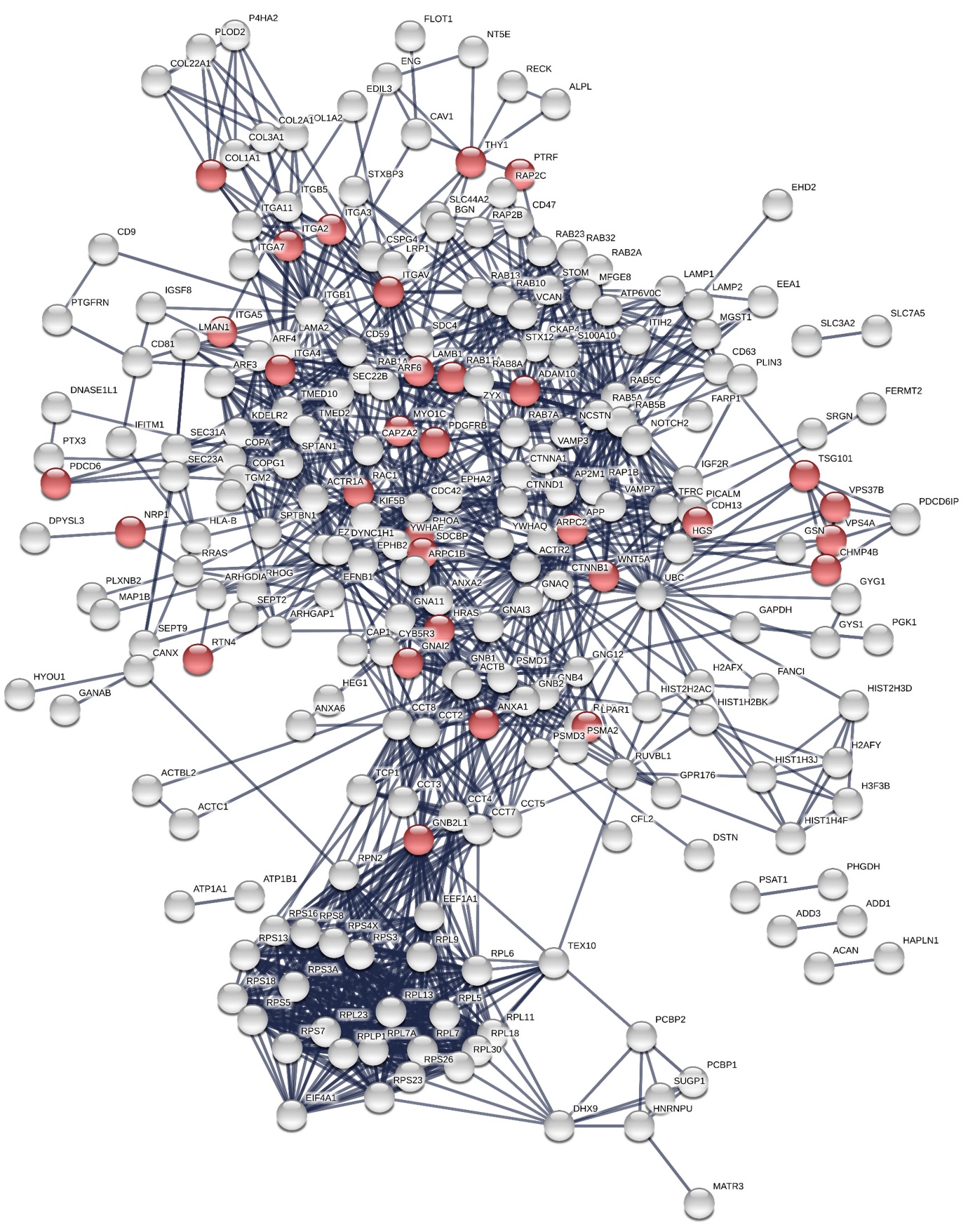


**Supplementary Figure 4**

**Supplementary Figure 5**


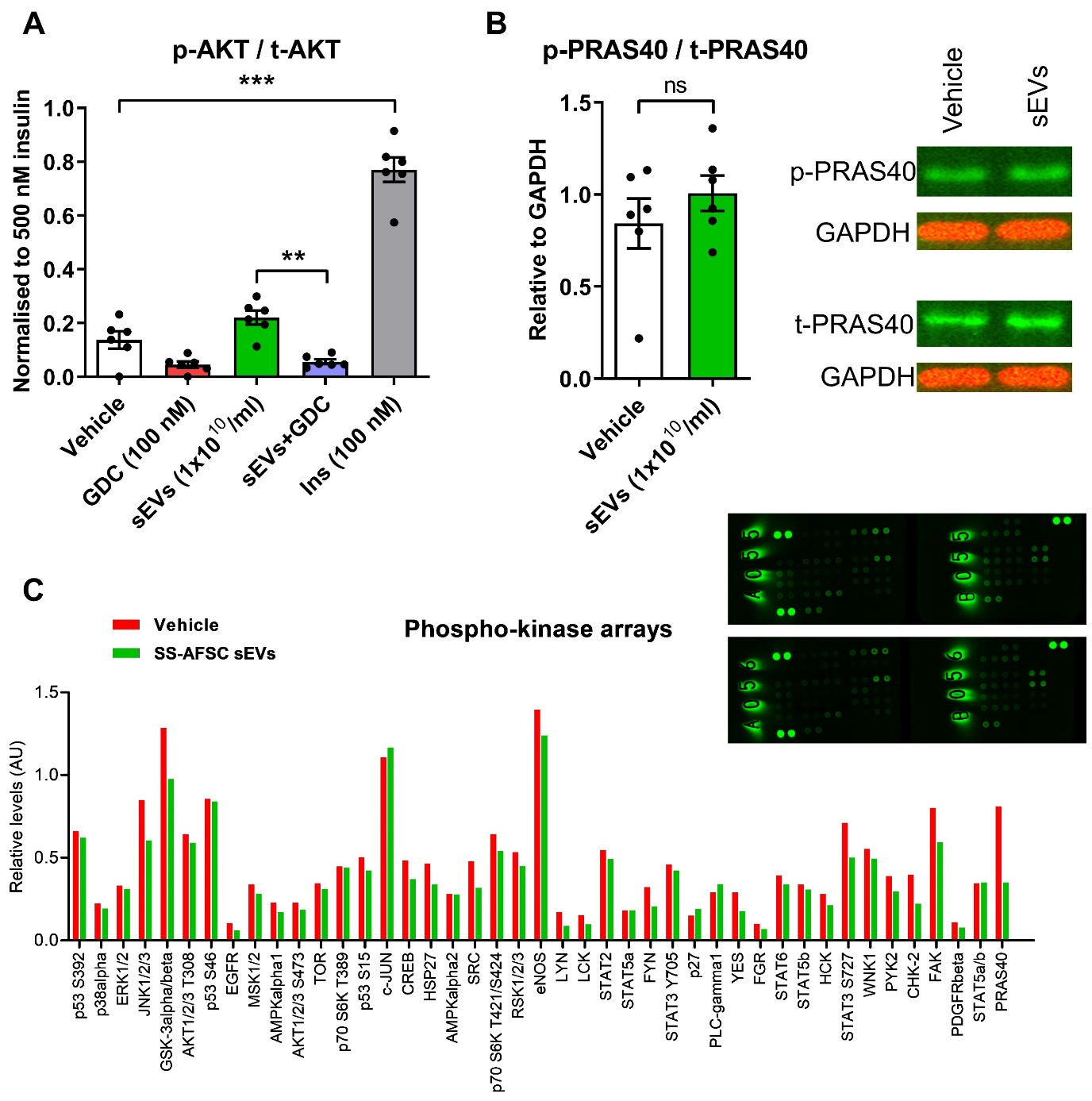


# Supplementary Figure Legends

**Supplementary Figure 1: SS-hAFSC expression of membrane markers**

SS-hAFSCs were analysed for MSC membrane marker expression using flow cytometry. Experiments for different MSC markers were performed and protein identities are indicated below graphs (isotype control also included). Grey peaks – unlabelled cells; red peaks – cells labelled for the indicated antigens.

**Supplementary Figure 2: SS-hAFSCs characteristics after incubation with or without serum**

A: Representative images of SS-hAFSC morphology in each condition. Scale: 200µm. B: Propidium iodide and annexin-V double staining of SS-hAFSCs for each condition followed by flow cytometry quantification. C: Results from B presented graphically. Cell apoptosis indicates propidium iodide-negative/annexin-V-positive cells as a percentage of total. n = 3. * p < 0.05, ns – non-significant (p > 0.05), 1-way Repeated Measures ANOVA with Tukey’s post-hoc test.

**Supplementary Figure 3: Establishment of a model of H_2_O_2_-induced death of primary cardiomyocytes**

A: Primary rat cardiomyocytes were incubated in the presence of increasing concentrations of H_2_O_2_ (0-125 µM) for 2 h. LDH release was measured at the end of the incubation period and presented as a percentage of total LDH. H_2_O_2_ dose-dependently increased cardiomyocyte death with an EC_50_ = 41.4 ± 4.1 µM (r^2^ = 0.8667). n = 6. B: Cardiomyocytes were treated with Vehicle (water) or increasing concentrations of N-acetylcysteine (NAC, 100-1000 µM) 30 min before addition of H_2_O_2_ and during the H_2_O_2_ incubation ([H_2_O_2_] = 40 µM). *** p < 0.001, * p < 0.05, ** p < 0.01, 1-way Repeated Measures ANOVA with Dunnett’s post-hoc test. LDH release calculated as per panel A. n = 4. C: Representative images of B. Scale: 200 µm.

**Supplementary Figure 4: STRING interaction network of proteins enriched in or exclusive to the SS-hAFSC sEVs**

Protein interaction network of SS-hAFSC sEV-enriched proteins (> 1.5 times) and SS-hAFSC sEV-exclusive proteins. Protein IDs are shown, and disconnected nodes are hidden. Proteins in red shading are associated with the Gene Ontology: Biological Process term “positive regulation of locomotion” (p = 2.7x10^-10^, 3.9-fold enrichment).

**Supplementary Figure 5: Activation of kinase pathways in endothelial cells in response to SS-hAFSC sEVs**

A,B: Western blotting results for total and phosphorylated AKT and PRAS40 in endothelial cells. In A: HUVECs were incubated with Vehicle (PBS, DMSO), GDC-0941 (100 nM) SS-hAFSC sEVs (1x10^10^ particles/ml), SS-hAFSC sEVs + GDC-0941 or insulin (100 nM, positive control) for 15 min. Results are presented relative to 500 nM insulin control. ** p < 0.01, *** p < 0.001, 1-way Repeated Measures ANOVA with Tukey’s post-hoc test. n = 6. p-AKT: phosphorylated AKT, t-AKT: total AKT. In B: HUVECs were incubated with Vehicle (PBS, DMSO) or SS-hAFSC sEVs (1x10^10^ particles/ml). Results are presented relative to GAPDH internal control. ns – non-significant (p > 0.05), Paired Student’s t-test. n = 6. p-PRAS40: phosphorylated PRAS40, t-PRAS40: total PRAS40. Representative images shown on the right. C: Phospho-kinase array for detection of phosphorylated kinases/kinase targets in HUVECs. Cells were incubated with vehicle (PBS) or SS-hAFSC sEVs (1x10^10^ particles/ml) for 3 h. Results are presented relative to control proteins. Images of the membranes shown in the top right corner: Vehicle (top, A055 and B055) and SS-hAFSC sEVs (bottom, A056 and B056).

# Supplementary Methods

Reagents were obtained from Sigma unless stated otherwise.

***Flow cytometry***

For SS-hAFSC characterisation, cells were collected and pelleted for 5 min at 500 g. Supernatant was removed and cells were washed once with 1% bovine serum albumin (BSA, Sigma) in PBS. Cells were then resuspended in 50 µl 1% BSA/PBS containing primary antibodies (1/25-1/10, Supplementary Table 1) and incubated on ice for 1 h in dark. If required, cells were washed and incubated with secondary antibody (1/50) for 1 h in dark (on ice) (Supplementary Table 1). Cells were then pelleted, washed once and resuspended in 300-500 µl 1% BSA/PBS for flow cytometry. 10,000 events were counted for each sample excluding debris on a BD Accuri™ C6 flow cytometer (BD Biosciences).

***In vivo non-recovery ischaemia/reperfusion injury model***

Sprague-Dawley rats (230-320 g) were anaesthetised with 100 mg/kg pentobarbital and placed in a supine position on a heating mat for maintaining a constant temperature of 36.5-37.5°C. Tracheostomy was performed, and artificial ventilation was achieved by means of a modified human intravenous 16 G catheter connected to a Small Animal Ventilator (Harvard Apparatus). Respiratory rate was set to 75 breaths/min, tidal volume was 8-9 ml/kg and expiratory tube was submerged in water to apply 2 cmH_2_O positive end-expiratory pressure. 1-lead electrocardiogram (ECG) was recorded using PowerLab/4SP system (AD Instruments) and LabChart 7 software. Jugular vein was cannulated using a 21 G butterfly needle for intravenous administration of substances.

Thoracotomy was performed, and an incision was made at the 4th intercostal space. 6-0 braided silk suture was positioned underneath the left anterior descending (LAD) artery and tightened. The presence of myocardial ischaemia was confirmed by myocardial blanching distal to the suture and ECG changes. After 30 min of ischaemia, reperfusion was achieved by releasing the suture. Treatments were administered 2 min prior to reperfusion as a single bolus intravenous dose (500 µl/animal, injected through the jugular vein cannula). Rats received vehicle (PBS), bradykinin (positive control for cardioprotection, 40 µg/kg; B3259, Sigma) or SS-hAFSC sEVs (2x10^11^ particles/animal, a dose resulting in approximately 1x10^10^ particles/ml blood).

After 2 h of reperfusion, the hearts were eviscerated, cannulated and washed with saline. The LAD artery was reoccluded and Evans Blue dye (1% w/v in saline) was injected to demarcate the non-risk area and the area at risk. The heart was then briefly frozen before sectioning and staining with triphenyl tetrazolium chloride (TTC, 1% w/v in phosphate buffer, pH 7.4) at 37°C for 15 min. Heart segments were then transferred to a 40% (v/v) formaldehyde solution and left overnight at room temperature for fixation. 2D images were obtained the next day using a CanoScan LiDE 220 scanner (Cannon) and ImageJ software was used to demarcate and calculate the non-risk area (defined as Evans Blue positive), the area at risk (defined as Evans Blue negative) and the infarct area (defined as Evans Blue negative and TTC negative). Area at risk was expressed as a percentage of the total left ventricle area. Infarct size was defined as the percentage of infarct area within the area at risk.

35 rats were used in total. 17 animals died during the experimental procedure (49%): 13 died prior to or unrelated to the administered treatments, 4 died post injection (2 in the Vehicle group, 1 in the Bradykinin group, and 1 in the sEV group). Hence, treatment-related death was not found in this experiment.

***Isolation of primary adult rat ventricular cardiomyocytes***

Buffer used for heart perfusion and cell collection (hereafter referred to as “Buffer”) contained 130 mM NaCl, 5.4 mM KCl (Fisher), 1.4 mM MgCl_2_ (Sigma), 0.4 mM Na_2_HPO_4_ (Sigma), 4.2 mM HEPES (Sigma), 10 mM glucose (Sigma), 20 mM taurine (Alfa Aesar) and 10 mM creatine (Sigma). The Buffer was maintained at 37°C and pH 7.4 during the isolation procedure.

Sprague-Dawley rats (250-350 g) were anaesthetised with 250 mg/kg pentobarbital (Animalcare). Upon loss of pedal reflex, thoracotomy was performed, and heart eviscerated. The aorta was immediately cannulated, and the heart was retrogradely perfused with Buffer containing 750 µM CaCl_2_ (Fluka). After a washout period, the heart was digested with Buffer containing 0.04-0.06% (w/w) collagenase type 2 (Worthington), 0.01% (w/w) protease (P5147, Sigma) and 100 µM CaCl_2_. The ventricles were excised, mechanically disrupted and the solution was sieved to remove fibrotic tissue and debris. Cardiomyocytes were collected with low speed centrifugation (< 100 g, 5 min). Extracellular Ca^2+^ was gradually restored using consecutive washes with Buffer containing 500 µM and 1 mM CaCl_2_ and finally cells were resuspended in Medium 199 (ThermoFisher) supplemented with 5 mM creatine, 2 mM carnitine (Sigma), 5 mM taurine, 50 units/ml penicillin (Sigma) and 50 µg/ml streptomycin (Sigma). Cardiomyocytes were seeded in 24-well plates on areas pre-coated with ~3-4 µg/cm^2^ laminin to facilitate cell adherence and stabilised overnight at 37°C / 5% CO_2_ before use for experiments.

***Hydrogen peroxide-induced cardiomyocyte death***

Primary cardiomyocytes were treated with H_2_O_2_ (Sigma) at increasing concentrations (1 µM – 125 µM) for 2 h to generate a dose-response curve. At the end of the treatment, medium was collected and refrigerated, and 1% Triton X-100 in PBS was added for 15 min to lyse the remaining live cells. Media and lysates were immediately processed for lactate dehydrogenase release assay (see below). Cardiomyocytes in normal conditions were used as a control.

To establish a positive control for the model, cardiomyocytes were pre-treated with 100–1000 µM N-acetyl-L-cysteine (NAC, Acros Organics) for 30 min. H_2_O_2_ was then added at 40 µM for 2 h (in the continuous presence of NAC). At the end of the experiment, supernatants were collected, cells lysed, and media and lysates processed as explained above. Cardiomyocytes in normal conditions were used as a control.

To investigate the SS-hAFSC sEV potential for protection against H_2_O_2_-induced cardiomyocyte death, cells were pre-treated with 1x10^10^ particles/ml SS-hAFSC sEVs for 30 min. H_2_O_2_ was then added at 40 µM for 2 h (in the continuous presence of sEVs). At the end of the experiment, supernatants were collected, cells lysed, and media and lysates processed as explained above. Cardiomyocytes in normal conditions were used as a control.

***Hypoxia/reoxygenation-induced cardiomyocyte death (simulated ischaemia/reperfusion in vitro)***

Primary cardiomyocytes were washed and culture medium was replaced with Hypoxic Buffer. Plates were transferred to a modular incubator chamber (Billups-Rothenberg) which was purged with 5% CO_2_ / 95% N_2_ to achieve hypoxic environment. The chamber was sealed and placed into an incubator at 37°C for 5 h. Hypoxic Buffer was then collected and refrigerated while cells were reoxygenated for 1 h in Normoxic Buffer to mimic reperfusion (Reoxygenation Buffer). At the end of the experiment, Reoxygenation Buffer was collected and refrigerated. 1% Triton X-100 in PBS was added for 15 min to lyse the remaining live cells. Hypoxic Buffer, Reoxygenation Buffer and cell lysates were immediately processed for lactate dehydrogenase release assay (see below). A control group with cardiomyocytes incubated in Normoxic Buffer was included.

For experiments assessing direct protection of cardiomyocytes, SS-hAFSC sEVs were added at 3x10^8^ – 3x10^9^ sEVs/ml 30 min prior to the hypoxic period and treatment continued throughout hypoxic and reoxygenation periods.

For experiments assessing indirect protection of cardiomyocytes, treatments were prepared as follows:

1) For Vehicle control group, vehicle (PBS) was incubated without any cells for 3 h in Endothelial Serum-Free Defined Medium (Cell Applications Inc; 113-500, Sigma) at 37°C / 5% CO_2_;

2) For sEV control group, 1x10^10^ SS-hAFSC sEVs/ml were incubated without any cells for 3 h in Endothelial Serum-Free Defined Medium (Cell Applications Inc; 113-500, Sigma) at 37°C / 5% CO_2_;

3) For HUVEC + Vehicle control group, HUVECs were incubated with vehicle (PBS) for 3 h in Endothelial Serum-Free Defined Medium (Cell Applications Inc; 113-500, Sigma) at 37°C / 5% CO_2_;

4) For HUVEC + sEV group, HUVECs were incubated with 1x10^10^ SS-hAFSC sEVs/ml for 3 h in Endothelial Serum-Free Defined Medium (Cell Applications Inc; 113-500, Sigma) at 37°C / 5% CO2;

For all groups, conditioned medium was collected and concentrated on Amicon Ultra-4 ultrafiltration units (3 kDa cut-off, regenerated cellulose membranes). Final concentrate volumes were recorded, and samples were frozen at -80°C.

To mimic more closely the *in vivo* experiment, treatments were administered at reoxygenation only. Treatments were normalised by area occupied by cells, *i.e.* conditioned medium from 6 area units occupied by HUVECs were added per 1 area unit occupied by cardiomyocytes (6:1 HUVEC to cardiomyocyte area proportion). This corresponded to ~15% of the medium conditioned by a confluent layer of HUVECs in a T75 flask per each well of the 24-well plate seeded with cardiomyocytes.

***Lactate dehydrogenase (LDH) assay***

LDH release was used as a surrogate measurement for cardiomyocyte death as its release in the extracellular milieu upon disruption of the cell membrane correlates with cell death^7^.

To account for differences in the starting number of cells in the H_2_O_2_-induced and hypoxia/reoxygenation-induced cell death assays, LDH release was normalised to the total amount of LDH (for H_2_O_2_ assay: LDH_total_ = LDH_medium_ + LDH_lysate_; for hypoxia/reoxygenation assay: in experiments assessing direct protection of cardiomyocytes: LDH_total_ = LDH_hypoxic_ + LDH_reoxygenation_ + LDH_lysate_ and in experiments assessing indirect protection of cardiomyocytes: LDH_total_ = LDH_reoxygenation_ + LDH_lysate_).

LDH was assayed using Pierce LDH Cytotoxicity Assay Kit (ThermoFisher) according to the manufacturer’s instructions with some modifications. 50 µl of each collected sample were added to a 96-well plate (25 µl diluted to 50 µl in case of experiments with higher cell numbers). 50 µl assay buffer were added to each well and the plate was shaken for 15 min in dark. After adding Stop Solution, the absorbance at 490 nm was measured on a FLUOstar plate reader (BMG Labtech). LDH release was presented as a percentage of total LDH.

***Endothelial cell migration***

Bottom wells of a 12-well NeuroProbe chemotaxis chamber (AA12, NeuroProbe) were filled with vehicle (PBS), 10% FBS (positive control), SDF-1α (positive control; 130-093-997, Miltenyi Biotec) or the indicated concentrations of SS-hAFSC-conditioned medium or SS-hAFSC sEVs. An 8-µm pore polycarbonate track-etch membrane (PFB8, NeuroProbe) was used as a barrier between top and bottom wells. 30,000 HUVECs/well in Endothelial Serum-Free Defined Medium (Cell Applications Inc; 113-500, Sigma) were plated in the top wells and the chamber was incubated for 6 h at 37°C / 5% CO_2_. At the end of the incubation period, membranes were collected, and the top side was scraped to remove non-migrated cells. Membranes were fixed in 100% cold methanol, stained using 0.5 % (w/v) Crystal Violet solution and scanned on CanoScan LiDE 220 scanner (Cannon). ImageJ was used to quantify the total staining intensity of each well. Intensities were measured for duplicate wells and presented as raw values or normalised to the positive control (10% FBS). For some experiments, pharmacological inhibitors (GDC-0941: SM19-10, Cell Guidance Systems at 100 nM; AMD3100: 3299, Tocris Bioscience at 10 µM; TAK-242: 614316, Calbiochem at 10 µM) or antibodies (anti-PTX3, Clone EPR18678-105, Abcam at 1 µg/ml; anti-RTN4B, Clone AF6034, R&D Systems at 1 µg/ml or 5 µg/ml) were added to both top and bottom wells of the chamber.

***Endothelial cell proliferation***

5,000 HUVECs/well were plated in a 96-well plate (~15,000 cells/cm^2^) in standard propagation medium (see above). Cells were allowed to attach for 2 h at 37°C / 5% CO_2_ followed by washing and replacement of medium with Endothelial Serum-Free Defined Medium (Cell Applications Inc; 113-500, Sigma) containing vehicle (PBS), 10% FBS (positive control) or SS-hAFSC sEVs at the indicated concentrations. Cells were incubated for 48 h and medium was exchanged once at 24 h. After the incubation period, 3-(4,5-dimethylthiazol-2-yl)-2,5-diphenyltetrazolium bromide (MTT) dissolved in PBS was added to a final concentration of 0.45 mg/ml. Plates were kept at 37°C / 5% CO_2_ for further 3 h to allow formation of formazan crystals from the MTT in the living cells. This was followed by removal of the supernatant, addition of lysis solution (0.1 M HCl / 10% Triton X-100 in propan-2-ol) and shaking for 10 min at 300 rpm. The absorbance at 570 nm and 690 nm was measured on a FLUOstar plate reader (BMG Labtech). The results are presented as A_570_-A_690_ and as a percentage of the positive control.

***Endothelial cell tube formation***

HUVEC tube formation assay was performed using a thin-layer extracellular gel matrix (i.e. Geltrex™, ThermoFisher)^8^. It is aimed at a significant reduction of resources and an optimised application for microscopy studies^8^.

Geltrex™ was thawed at 4°C and kept on ice during pipetting. 2 µl gel was pipetted in each well of a 96-well plate. The gel was evenly distributed in the well by the use of an insert of a tip for a repeater pipette. This was performed on ice to minimise gel solidification due to exposure to higher temperatures. The plate was then transferred to a cell culture incubator at 37°C for ~1 h to ensure Geltrex™ is completely solidified.

HUVECs were starved for 1-1.5 h in Endothelial Cell Basal Medium 2 (PromoCell) supplemented with 1% FBS (without growth factors to eliminate interference with tube formation). Cells were then collected, resuspended and pipetted into wells covered with Geltrex™ at 10,000 cells/well. Treatments were added at the indicated concentrations and plates were incubated for 16 h in the incubator at 37°C / 5% CO2. After the incubation period, wells were washed with PBS and fixed using 4% (w/v) formaldehyde solution. Images were taken using Nikon Eclipse TE200 inverted microscope (Nikon). Tube-like structures were manually counted using ImageJ software.

***Protein arrays – phosphorylated kinases***

Phosphorylation of intracellular signalling kinase pathways was investigated using Proteome Profiler Human Phospho-Kinase Array Kit (ARY003B, R&D Systems) following manufacturer’s instructions. HUVECs were pre-starved for 3 h in DMEM (ThermoFisher) supplemented with 25 mM glucose, 4 mM GlutaMAX, 50 units/ml penicillin (Sigma), 50 µg/ml streptomycin (Sigma). Cells were then treated with vehicle (PBS) or sEVs (1x10^10^ particles/ml) for 15 min or 3 h, as indicated, followed by immediate *in situ* lysis using the buffer provided in the kit supplemented with protease inhibitors (1861279, ThermoFisher). Lysates were rocked for 30 min at 4°C for complete breakdown of the cells and centrifuged for 5 min at 14,000 g. Pellets were discarded and the supernatant collected and stored at -80°C. ~300 µg protein was loaded on each membrane set. Streptavidin-DyLight 800 conjugate was used for detection of biotin-conjugated antibodies at 250 ng/ml (21851, ThermoFisher). Membranes were imaged, and densitometry was performed on Odyssey system. The spot coordinates can be found on <https://resources.rndsystems.com/pdfs/datasheets/ary003b.pdf>. Duplicate spot pixel densities were normalised to control protein spots for each membrane and presented as relative pixel densities.

***Western blotting***

HUVECs were pre-starved for 3 h in DMEM (ThermoFisher) supplemented with 25 mM glucose, 4 mM GlutaMAX, 50 units/ml penicillin (Sigma), 50 µg/ml streptomycin (Sigma). Cells were then treated for 15 min with vehicle (PBS), insulin (positive control at 100 nM or 500 nM as indicated; I9278, Sigma) or sEVs (3x10^9^ or 1x10^10^ particles/ml as indicated). GDC-0941 (100 nM, SM19-10, Cell Guidance Systems), a PI3K-specific inhibitor, was added 10 min prior to administration of vehicle/sEVs. Following the treatments, cells were immediately lysed *in situ* using a buffer consisting of 100 mM Tris base (Sigma), 300 mM NaCl (Sigma) and 0.5% NP-40, pH 7.4 with added protease inhibitors (1861279, ThermoFisher) and phosphatase inhibitors (78427, ThermoFisher). Debris was discarded by 10 min centrifugation at 10,000 g and the protein-rich supernatant was stored at -80°C.

20 µg protein (as measured by a BCA assay) of each sample was loaded in a well of a 10% Tris-Glycine pre-cast gel (XP00105BOX, ThermoFisher). This was followed by electrophoresis using 25 mM Tris base (Sigma)/250 mM glycine (Sigma)/0.1% SDS (Sigma) buffer and wet transfer of proteins to nitrocellulose membrane (10600003, GE Healthcare). The membranes were then blocked using a solution of 5% BSA in PBS and incubated with primary antibodies in 5% BSA/0.1% Tween-20/PBS overnight at 4°C (see primary antibody details in Supplementary Table 3). Secondary antibodies were added for 1 h at room temperature (1/10,000; goat anti-mouse IgG, 926-32210, LI-COR and goat anti-rabbit IgG, 926-32211, LI-COR). Membranes were imaged, and densitometry was performed on Odyssey system (LI-COR). Results are presented relative to 500 nM insulin positive control or GAPDH internal reference.

# Supplementary Tables

**Supplementary Table 1** – Antibodies used for flow cytometry analysis of SS-hAFSCs

|  | Antigen | Conjugate | Clone / Cat no | Company |
| --- | --- | --- | --- | --- |
| 1ary | CD14 | PE | TÜK4 | Miltenyi Biotec |
|  | CD29 | PE | MAR4 | BD Biosciences |
|  | CD34 | FITC | 581 | BD Biosciences |
|  | CD44 | - | J-173 | Abcam |
|  | CD45 | FITC | HI30 | BD Biosciences |
|  | CD73 | PE | AD2 | Miltenyi Biotec |
|  | CD90 | APC-Vio770 | DG3 | Miltenyi Biotec |
|  | CD105 | FITC | 43A4E1 | Miltenyi Biotec |
|  | IgG (isotype control) | PE | IC002P | R&D Systems |
| 2ary | Mouse IgG | Alexa Fluor 488 | ab150117 | Abcam |

**Supplementary Table 2** – Composition of Normoxic and Hypoxic buffers used for *in vitro* hypoxia/reoxygenation experiments with primary rat cardiomyocytes

**Normoxic Buffer**

| Substrate | Final conc. (mM) |
| --- | --- |
| Glucose | 10.0 |
| NaCl | 116.0 |
| KCl | 2.6 |
| KH_2_PO_4_ (Fisher) | 1.2 |
| MgSO_4_ x 7H_2_O (Acros Organics) | 1.2 |
| NaHCO_3_ (Fisher) | 24.0 |
| CaCl_2_ | 1.8 |

**Hypoxic Buffer**

| Substrate | Final conc. (mM) |
| --- | --- |
| NaCl | 136.6 |
| KCl | 14.8 |
| KH_2_PO_4_ | 1.2 |
| MgSO_4_ x 7H_2_O | 1.2 |
| NaHCO_3_ | 3.4 |
| CaCl_2_ | 1.8 |

**Supplementary Table 3** – Antibodies used for Western blotting analysis of signalling pathways in HUVECs

| Antigen | Species | Cat no | Company |
| --- | --- | --- | --- |
| total-AKT | Mouse | 2920 | Cell Signalling Technology |
| phospho-S473-AKT | Rabbit | 4060 | Cell Signalling Technology |
| total-PRAS40 | Rabbit | 2610 | Cell Signalling Technology |
| phospho-T246-PRAS40 | Rabbit | 2997 | Cell Signalling Technology |
| GAPDH | Mouse | sc-32233 | Santa Cruz Biotechnology |
